# Efficacy of the Mediterranean diet in treating metabolic dysfunction-associated steatotic liver disease (MASLD) in children and adolescents: a systematic review and meta-analysis

**DOI:** 10.1186/s12889-024-19378-w

**Published:** 2024-10-03

**Authors:** Asma Jamil, Tawanda Chivese, Usra Elshaikh, Marguerite Sendall

**Affiliations:** 1 Department of Public Health, College of Health Sciences, QU Health, Qatar University, Doha, Qatar; 2 Department of Population Medicine, College of Medicine, QU Health, Qatar University, Doha, Qatar; 3 Department of Public Health , Canadian University Dubai, Dubai, United Arab Emirates

**Keywords:** Non-alcoholic fatty liver disease, Pediatric, Children, Mediterranean diet, MASLD

## Abstract

**Background:**

There are limited treatment options for metabolic dysfunction-associated steatotic liver disease (MASLD), formerly known as non-alcoholic Fatty Liver Disease (MASLD) in children and adolescents.

**Aim:**

To evaluate the effectiveness of the Mediterranean diet in improving liver function in children and adolescents with MASLD.

**Methods:**

In this systematic review and meta-analysis, we searched PubMed, Scopus, Embase, CINAHL, and Cochrane CENTRAL for interventional studies investigating the effect of Mediterranean diet on MASLD in children and adolescents. The primary outcome was a change in liver function measured using these liver enzymes; Alanine Transaminase (ALT), Aspartate Transaminase (AST) and Gamma-glutamyl transferase (GGT). The secondary outcomes were lipid profile, body weight, and insulin resistance. The risk of bias was assessed using the MASTER scale. Bias-adjusted inverse variance heterogeneity models were used to synthesize overall weighted mean differences for the treatment effect (WMD) and their 95% confidence intervals. Heterogeneity and publication bias were evaluated using the I^2^ statistics, Tau-squared and Doi plots, respectively.

**Result:**

Out of 5915 study records identified from database searches, five studies with 308 participants, two randomized controlled trials, and three quasi-experimental studies, met the inclusion criteria. In overall synthesis, the Mediterranean diet was associated with moderate improvements in liver function as shown by reductions in the liver enzymes [ALT - WMD − 10.85 U/L, 95% CI -20.03 to -1.68, I^2^ = 42, *T*^*2*^ = 38.8, AST - WMD − 9.26 U/L, 95% CI -17.14 to -1.38, I^2^ = 70.7, *T*^*2*^ = 42.7, and GGT - WMD − 1.99 95% CI -5.09 to 1.11)], but changes in body weight, lipid profile and insulin resistance were small and insignificant.

**Conclusion:**

The Mediterranean diet may improve liver function in children with MASLD. More randomized controlled trials are needed to develop high-certainty evidence on these findings.

**Registration:**

This protocol was registered on the International Prospective Register of Systematic Reviews (PROSPERO) CRD42023426939. 31/05/2023.

**Supplementary Information:**

The online version contains supplementary material available at 10.1186/s12889-024-19378-w.

## Introduction

Metabolic dysfunction-associated steatotic liver disease (MASLD), formerly known as nonalcoholic fatty liver disease is a long-term condition that is multifaceted in nature [1]. MASLD occurs when there is fat accumulation in more than 5% of hepatocytes, with no evidence of alcohol intake, viral hepatitis, or drug-induced liver steatosis [1]. MASLD is closely associated with obesity and has become the most common liver disease in children in the United States [1]. In the last 10 years, MASLD has become most common reason for liver transplants in adults [1]. The prevalence of MASLD has increased over recent years from 2000 per 100,000 person-years in the year 2000 to 7000 per 100,000 in the year 2015 [2]. MASLD is associated with increased risk of type 2 diabetes, and mortality from cardiovascular diseases [3–5].

Presently, there is no approved pharmacological treatment to treat MASLD [1]. Recent MASLD guidelines published by the European Society for Clinical Nutrition and Metabolism in 2020, recommend Mediterranean diet to reduce steatosis and improve insulin sensitivity. Adherence to the Mediterranean diet has shown reduced severity of MASLD [4]. However, these guidelines are based on adult populations and did not include children and adolescents [6]. The guidelines published by North American Society for Pediatric Gastroenterology, Hepatology, and Nutrition (NASPGHAN) and the American Association for the Study of Liver Diseases (AASLD) have no recommendations regarding any particular diet to treat MASLD because there is not enough evidence to support a specific diet. The current recommendations include avoiding sugar-sweetened beverages and consuming a balanced diet [1, 7] which are part of the Mediterranean diet.

Meta-analyses conducted in adults have shown promising results of Mediterranean diet on MASLD [3, 8]. Several studies have demonstrated the mitigatory effect of the Mediterranean diet on cardiovascular disease, cognition, cancer, and diabetes [6, 9, 10]. Recently, several systematic reviews and meta-analyses have also demonstrated a positive impact of the Mediterranean diet in treating MASLD in adults [3, 8, 11].

Many meta-analyses have been published on the effectiveness of the Mediterranean diet on liver biomarkers in adults [3, 12]. These studies have found the Mediterranean diet is effective in improving one or more liver enzymes and improving indirect outcomes such as weight loss, insulin sensitivity and lipid profile. In one review, the findings indicated that even when the Mediterranean did not result in weight loss, some study participants showed improvements in biomarkers [3]. However, there are limitations to these findings. Both meta-analyses showed high heterogeneity, attributed to either small sample size, different ways of measuring MASLD severity, or variation in the control diet [12]. Recent research has shown that the Mediterranean diet might have a beneficial effect on children and adolescents with MASLD. However, the precise benefit on liver biomarkers and the benefit of the Mediterranean diet compared to other diets remains unclear [5, 9, 13]. It is important to conduct this systematic review and meta-analysis as there is a lack of evidence-based guidelines to recommend the best dietary intervention to treat MASLD in children and adolescents and because of the differing effectiveness of the Mediterranean diet on liver biomarkers compared to other diets. The aim of this study was to synthesise the current evidence on the effectiveness of the Mediterranean diet compared to alternative diets on MASLD in children and adolescents.

## Methods

### Study design

This study is a systematic review and meta-analysis following the PRISMA guidelines [14]. The protocol was registered in the International Prospective Register of Systematic Reviews (PROSPERO: CRD42023426939).

### Data sources

A comprehensive search was conducted from 19th to 27th August 2023 on databases. Electronic data bases such as Cochrane Central Register of Controlled Trials (CENTRAL), PubMed, Scopus, Embase, Cumulated Index to Nursing and Allied Health Literature (CINAHL), and database of preprints (medRXIV) were searched for published studies. The ClinicalTrials.gov and WHO’s International Clinical Trials Registry Platform (ICTRP), and Dissertations and Theses Global were searched for grey literature. Elicit AI and SearchRabbit were searched to find published articles and grey literature along with backward and forward citation checks.

### Search strategy

The search strategy was developed through a review of meta-analyses of topic in the same subject and developing and refining keywords from the concepts in the PICO question. In PubMed we used both keyword searched and MeSH terms. MeSH terms for MASLD, Mediterranean diet, children and adolescents were identified from the MeSH database. A detailed search strategy is provided in Supplemental Table 2.

### Screening process of studies

The titles and abstract of retrieved articles were transferred to EndNote 20 for deduplication before preliminary screening on the Rayyan systemic review management website (https://www.rayyan.ai/). After screening titles and abstracts, full texts of data sources were manually reviewed for eligibility by two independent reviewers (AJ and UE).

### Eligibility criteria

All experimental and interventional studies studying the effect of the Mediterranean diet on MASLD in children and adolescents were included with no language and time restriction.

### Inclusion criteria

Studies conducted on children and adolescents from 2 to 19 years of age with a confirmed MASLD diagnosis who have not had a liver transplant. The intervention (Mediterranean diet) could be dietary advice, provision of relevant foods, or both. Included studies should have reported the primary outcome of interest, that is liver enzymes. If a study reported changes in liver enzymes as secondary outcomes they were included if the intervention was the Mediterranean diet.

### Exclusion criteria

Studies conducted on adult patients only were excluded. Studies conducted on animals were excluded. Studies only studying adherence to Mediterranean diet were excluded. Systemic reviews, narrative and literature reviews, and observational studies were also excluded.

### Data extraction/data synthesis process

All eligible full-text articles were screened by two reviewers (AJ and UE) and data was extracted using a detailed data extraction form on Excel. These two authors (AJ and UE) checked one article by extracting data and comparing extracted information. The following data were extracted from the selected articles: study design, population characteristics, country of origin, year of publication, age, gender, total number of participants, intervention and control diet composition, and primary and secondary outcomes described below. Disagreements during screening were resolved through critical discussion. If these two authors could not reach an agreement, the second and fourth (TC, MS) were consulted, and a final decision was made.

### Primary outcomes

The primary efficacy outcome was change in ALT, a surrogate marker for the improvement of MASLD in children and adolescents as mentioned in the guidelines [1]. Changes in the levels of AST, GGT were evaluated even though they are not accurate indicators of liver improvement because increases in AST and GGT are linked to poor histology in the presence of elevated ALT. All liver enzymes were measured in U/L.

### Secondary outcomes

Secondary outcomes included the lipid profile, insulin resistance and body weight change. The lipid metabolism was evaluated using total cholesterol, LDL-cholesterol, HDL-cholesterol, and triglyceride levels. Lipid parameters were measured in mg/dl. Homeostatic Model Assessment for Insulin Resistance (HOMA-IR) was also evaluated. Change in body weight was measured in kg.

### Study risk of bias / quality assessment

The quality of studies was assessed using The MethodologicAl STandards for Epidemiological Research (MASTER) scale [15]. It is applicable to multiple analytical study designs. This tool has 36 items included in 7 quality domains which are equal recruitment, equal retention, equal ascertainment, equal implementation, equal prognosis, sufficient analysis, and temporal precedence. Disagreements were resolved by discussion with the authors (MS and TC).

### Synthesis methods

#### Synthesis of results

Mean difference (MD) and standard deviation (SD) of liver enzymes and other outcomes of interest were extracted from studies for intervention and control groups using a detailed formula workbook on Excel. A bias-adjusted inverse variance heterogeneity meta-analysis (quality effects model) was used to compute a weighted mean difference (WMD) and 95% confidence interval (CI) HOMA-IR, total cholesterol, LDL, HDL, body weight as they are continuous and measured on the same scale. One of the studies [5] reported median and inter-quartile range without Q1 and Q3 for liver enzymes and HOMA-IR. It was assumed the distribution of these variables was symmetric and IQR was divided by 2 to get Q1 and Q3, they were then entered into a detailed Excel formula sheet [16] to ascertain mean and SD. The included studies included values for baseline and end-of-treatment for intervention and control groups. To establish final values for each groups, we calculated the mean change-from-baseline, change score, using the formula, Mean (change) = Mean (Endpoint) - Mean (Baseline). The change in standard deviation was calculated following steps from the Cochrane Handbook for Systematic Reviews of Interventions [17]. Another study [18], reported mean and 95% CI. This was converted to mean and SD using the formula SD = $\:\sqrt{\text{n}}$ x (upper limit – lower limit 95% CI)/3.92 [19]. To retain the independence of data and avoid counting participants twice in the meta-analysis of before and after studies, we conducted individual analyses of each study to establish the mean difference and 95% CI of this difference using direct tests. We had two kinds of data, one was from studies that had change of score studies which had before and after values for treatment and control groups [5, 13, 20], for these we calculated the mean difference and difference of standard deviation. We used the variance test to establish variance which was used in the t-test to establish mean difference and its 95% CI. The other type of studies [18, 21] had data for before and after studies, without a separate control group, one of these studies reported the mean and 95% CI which we changed to SD. Since there was no direct way to calculate 95% CI for paired/dependent studies which only reported mean, standard deviation, and sample size without reporting the correlation coefficient, we approximated the standard deviation of the differences using summary statistics such as means, standard deviations, and sample sizes. First, we calculated the standard error (SE) of mean differences using the below formula, where N is the total sample size.

$$S{E_{diff}} = \sqrt {{{SD_{{\rm{before}}}^2} \over N} + {{SD_{{\rm{after}}}^2} \over N}}$$

Then we used the below formula to calculate the standard deviation of the differences,

$$S{D_{diff}} = S{E_{diff}} \times \sqrt N$$

We then calculate the margin of error (ME) using,

$$ME = {t_{\alpha /2}} \times S{E_{diff}}$$

We used = tinv (p-value, degree of freedom) in excel and multiplied it with SE of difference. We could establish the 95% CI of difference using,

$$CI = \left( {{\rm{Mean}}\,{\rm{difference}} - ME,\,{\rm{Mean}}\,{\rm{difference}} + ME} \right)$$

These values were used to do a meta-analysis analysing the effect sizes. The quality effects model (QE) takes into account heterogeneity and the quality of the study [22]. This model differs from the random effects (RE) model in the way it adjusts for heterogeneity and further adjusts for bias when weighting studies in a meta-analysis [22]. However, as the RE is the most commonly used model in meta-analyses, we have also presented the syntheses of the RE model for comparison and to show prediction intervals of the study outcome variables. The quality scores used are shown in Supplemental Table 1. Heterogeneity was assessed and quantified with Cochran’s Q, Tau-squared (T^2^), and I^2^ (lower values indicate lower heterogeneity). Galbraith plot was used to identify the outlying studies and sensitivity analysis was conducted to test the robustness of the findings. Publication bias was assessed using Doi plots and funnel plots. *Metan* in Stata version 18 (College Station, Texas, USA) was used to conduct the meta-analysis.

## Results

### Description of the search

**Fig. 1:**
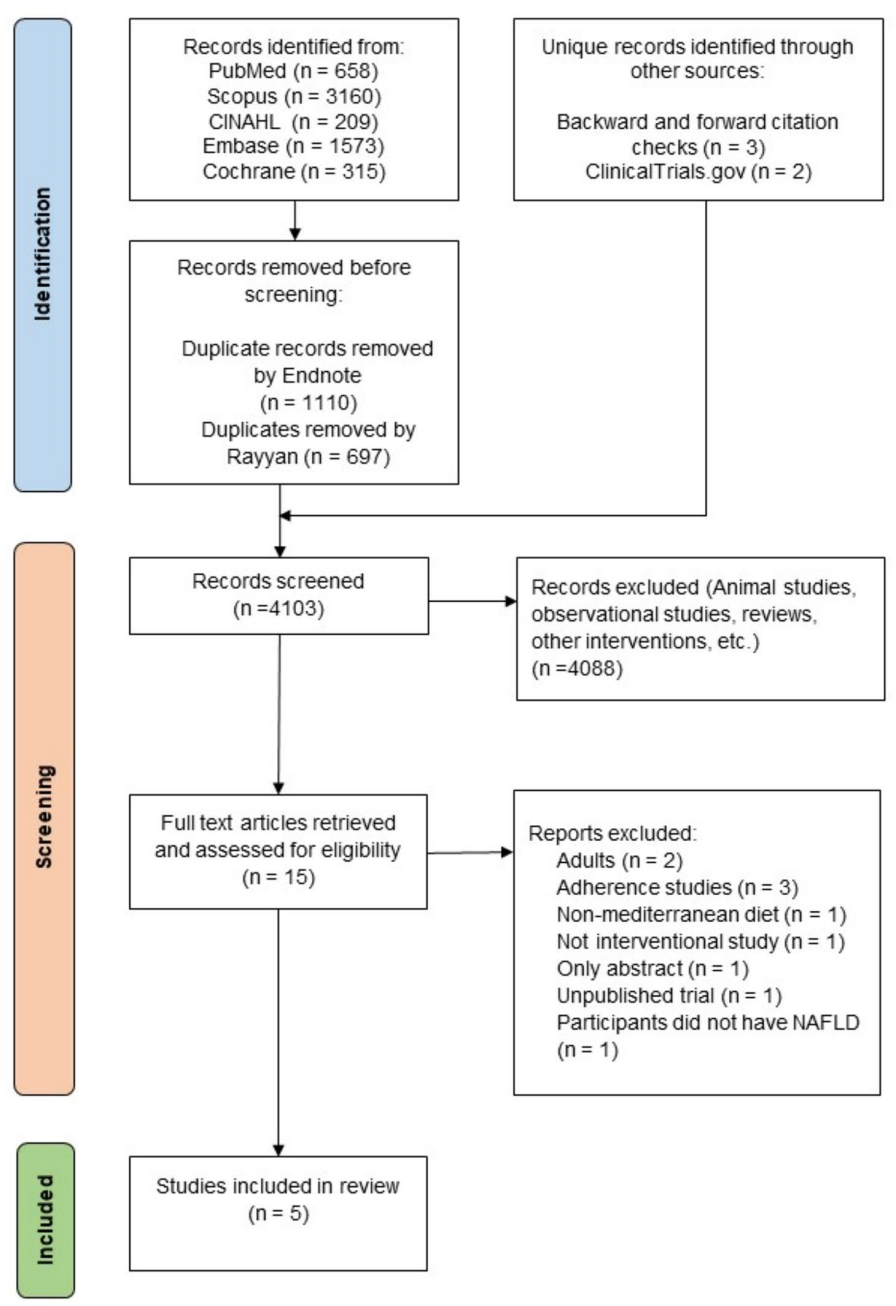
PRISMA flow diagram

The database search identified 5915 study records. After the removal of duplicates and screening a total of 15 study records were identified and then assessed for eligibility using full text. Ten studies were excluded for the reasons in Fig. 1. A total of 5 studies were included in the meta-analysis.

### Characteristics of included studies

Table 1 shows the characteristics of the five included studies. Two of the studies were randomized controlled trials [5, 13] from Turkey. The comparator group in both trials was the low-fat diet. Among the three remaining quasi-experimental studies, one was conducted in Poland [20]. All the participants in the study were asked to follow the Mediterranean diet. Those who had a decrease in BMI and were 100% compliant to the diet were considered as the intervention group, and those who did not follow the regimen were considered the control group. Two of the studies [18, 21] were conducted in Italy. Both had the same study design; liver function was assessed at baseline and after the end of a lifestyle change intervention which consisted of the Mediterranean diet and physical activity.

**Table 1:** Characteristics of included studies

Author	Population	Study duration Sex (Male) Mediterranean diet/ Control	Study design	Age years, mean ± SD, Mediterranean diet/ Control	Total number of participants Mediterranean diet/ Control	Intervention	Mediterranean diet	Control
Yurtdas, 2022 [5]	Turkey, adolescents with obesity and MASLD	2.7 months 13/13	Randomized controlled trial	13 ± 1.99 / 13.9 ± 2.34	22/22	Mediterranean diet	Mediterranean diet: 40% carbohydrates, 35–40% fat (< 10% saturated fat), 20% protein. Fish, legumes 2–3 times/week, daily walnuts (20 g/day), and olive oil (30–45 g/day)	Low-Fat diet: 50–60% carbohydrates, < 30% fat (with < 10% saturated fat), and 20% protein
Akbulut, 2022 [13]	Turkey, children overweight or obese with MASLD	2.7 months 17/15	Randomized controlled trial	12.9 ± 2.5 / 13.1 ± 2.5	30/30	Mediterranean diet and physical activity	Mediterranean diet: 40–44% carbohydrate, 35–40% fat (< 10% of saturated fat), 20% protein	Low-Fat diet: 55% carbohydrates, 20–25% fat (< 10% of saturated fat), and 20–25% protein
Malecki, 2021 [20]	Poland, children with MASLD	29 months 15/13	Quasi-experimental	NA / NA	22/20	Mediterranean diet and Physical activity	5 fruits/veggies daily, fish 2–3 times/week, olive oil, grains/dairy for breakfast, nuts 2–3 times/week, pulses weekly, pasta/rice daily, cut red meat, sugar, and sugary drinks	Regular diet (not adherent to Mediterranean diet)
Pacifico, 2013 [18]	Italy, children with obesity and MASLD	12 months 65/65	Quasi-experimental (before and after intervention study)	11.9 ± 4.629 / 11.9 ± 4.629	NA	Mediterranean diet: Italian RDA, Hypocaloric	25–30 kcal/Kg/day, 50–60% carbohydrates, 23–30% fat (2/3 unsaturated, 1/3 saturated), 15–20% protein, ω-6/ω-3 (4:1 ratio) including 5 whole grains high-fiber, 3 veggies, 2 fruits, 2–3 protein, 2–3 low-fat servings daily	Diet before intervention
Nobili, 2006 [21]	Italy, children with MASLD	12 months 57/57	Quasi-experimental (before and after intervention study)	11.7 ± 3.3 / 11.7 ± 3.3	NA	Mediterranean diet: Italian RDA, Hypocaloric	25–30 kcal/kg/d; carbohydrate, 50-60%; fat, 23-30%; protein, 15- 20%; fatty acid, two thirds saturated, one third unsaturated; ω-6/ω-3 (4:1 ratio)	Diet before intervention

### Assessment of study quality

The MASTER tool was used to assess the study quality in both the RCTs and the quasi-experimental studies. A total of 5 studies were included and the score ranged from 26 to 32 out of a total of 36. The RCTs scored the highest amongst the included studies. Quasi-experimental studies lacked in the domain of equal ascertainment and equal prognosis. The quality of the included studies is shown in Supplemental Table 1.

### Effect of the Mediterranean diet on liver enzymes: ALT

**Fig. 2:**
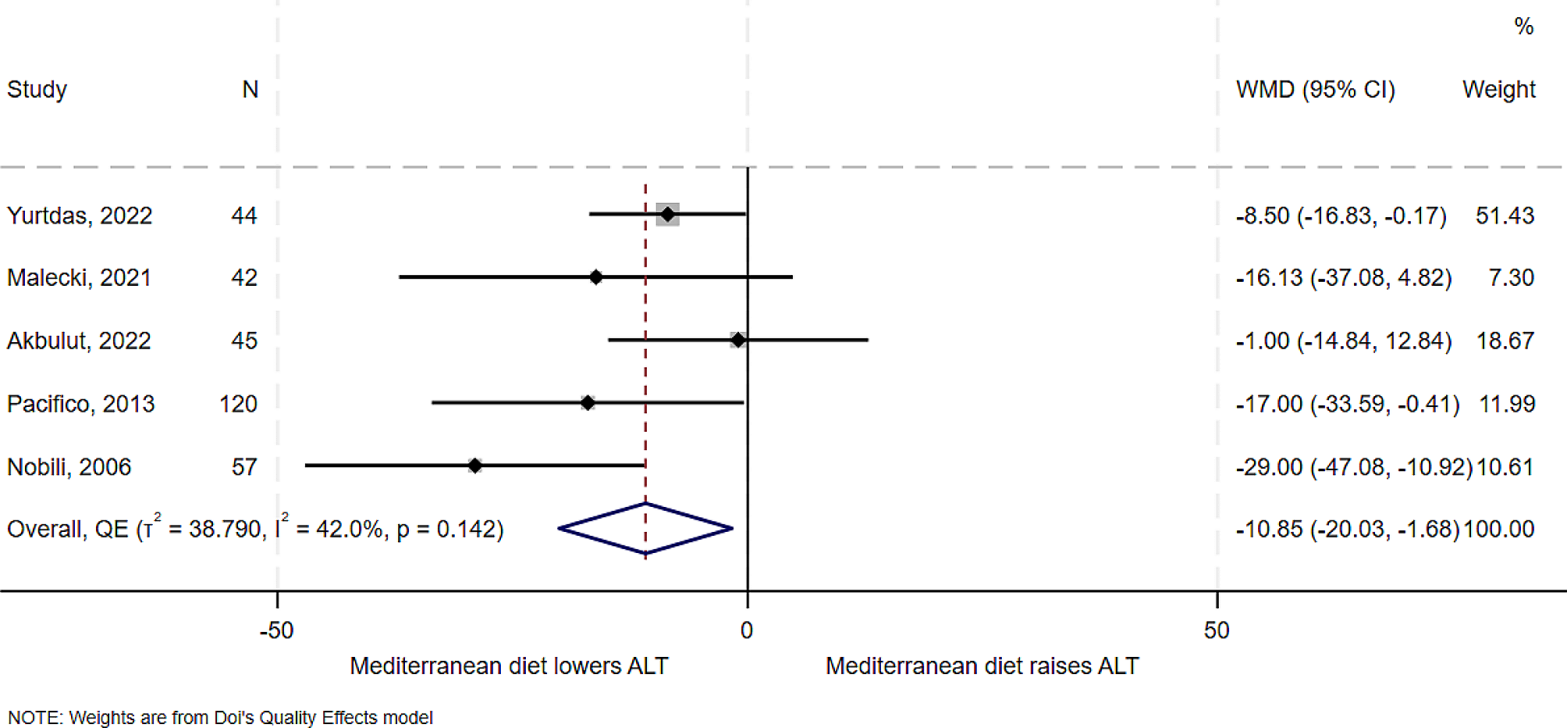
Forest plot showing the effect of the Mediterranean diet compared to other diets on ALT

All the five studies examined the effect of Mediterranean diet on ALT. Results from the studies showed variable effects from a mean decrease of 29 U/L in a before and after quasi-experimental study of Italian children of mean age 11.7 years [21] to a mean decrease of 1 U/L in an RCT of Turkish children of mean age of 13 years [13]. In overall synthesis (Fig. 2), the estimated effect of the Mediterranean diet was a mean reduction of 11 U/L in the level of ALT (WMD − 10.85, 95% CI -20.03 to -1.68), with low heterogeneity but high between study variance (I^2^ = 42%, *T*^*2*^ = 38.8), with no outlier on the Galbraith plot (Supplemental Fig. 1). There was some evidence of publication bias for this outcome (Supplemental Fig. 2).

### Effect of the Mediterranean diet on liver enzymes – AST

**Fig. 3:**
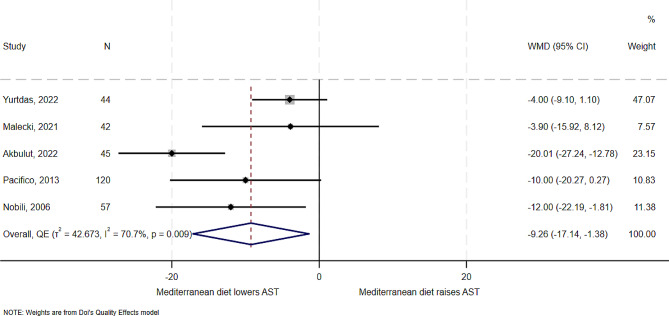
Forest plot showing the effect of the Mediterranean diet compared to other diets on AST

All five studies examined the effect of the Mediterranean diet on AST. Results from these studies showed variable effects from a mean decrease of 20 U/L in an RCT of Turkish children of mean age of 13 years [13] to a mean decrease of 3.9 U/L in an unrandomized quasi-experimental study of Polish children with mean age of 10.51 years [20]. In overall synthesis (Fig. 3), the estimated effect of the Mediterranean diet was a mean reduction of -9 U/L of AST (WMD − 9.26, 95% CI -17.14 to -1.38), with substantial heterogeneity (I^2^ = 70.7%, *T*^*2*^ = 42.7), with one study [13] being an outlier on the Galbraith plot (Supplemental Fig. 3). In analysis without the outlying study [13], the Mediterranean diet still showed benefit in reducing AST (WMD − 6.03, 95% CI -9.96 to -2.09), with low heterogeneity and variance between studies (I^2^ = 0%, *T*^*2*^ = 0) (Supplemental Fig. 4). There was some evidence of publication bias for this outcome (Supplemental Fig. 5).

### Effect of the Mediterranean diet on liver enzymes - GGT

**Fig. 4:**
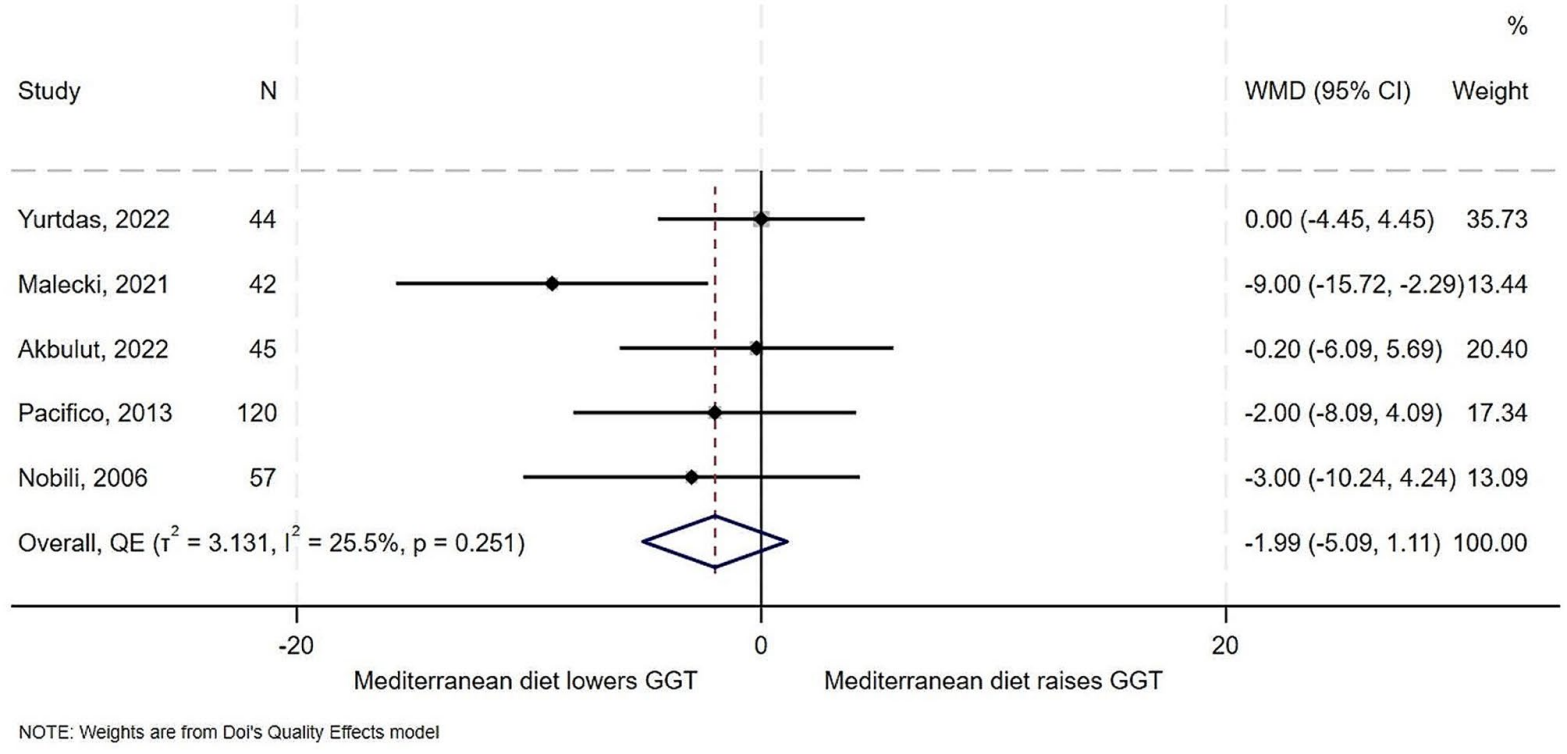
Forest plot showing effect of the Mediterranean diet compared to other diets GGT

All five studies examined the effect of the Mediterranean diet on GGT. Results from the studies showed variable effects from a mean decrease of 9 U/L in a control and treatment study of Polish children of mean age of 10.51 years [20] to a no effect of in an RCT of Turkish children of mean age of 13 years [5]. In overall synthesis (Fig. 4) the estimated effect of the Mediterranean diet was a decrease of 2 U/L in the level of GGT (WMD − 1.99, 95% CI -5.09 to 1.11), low heterogeneity (I^2^ = 25.5%, *T*^*2*^ = 3.1), on the Galbraith plot showed no outliers (Supplemental Fig. 6). There was some evidence of publication bias for this outcome (Supplemental Fig. 7).

### Effect of Mediterranean diet on insulin resistance-HOMA-IR

**Fig. 5:**
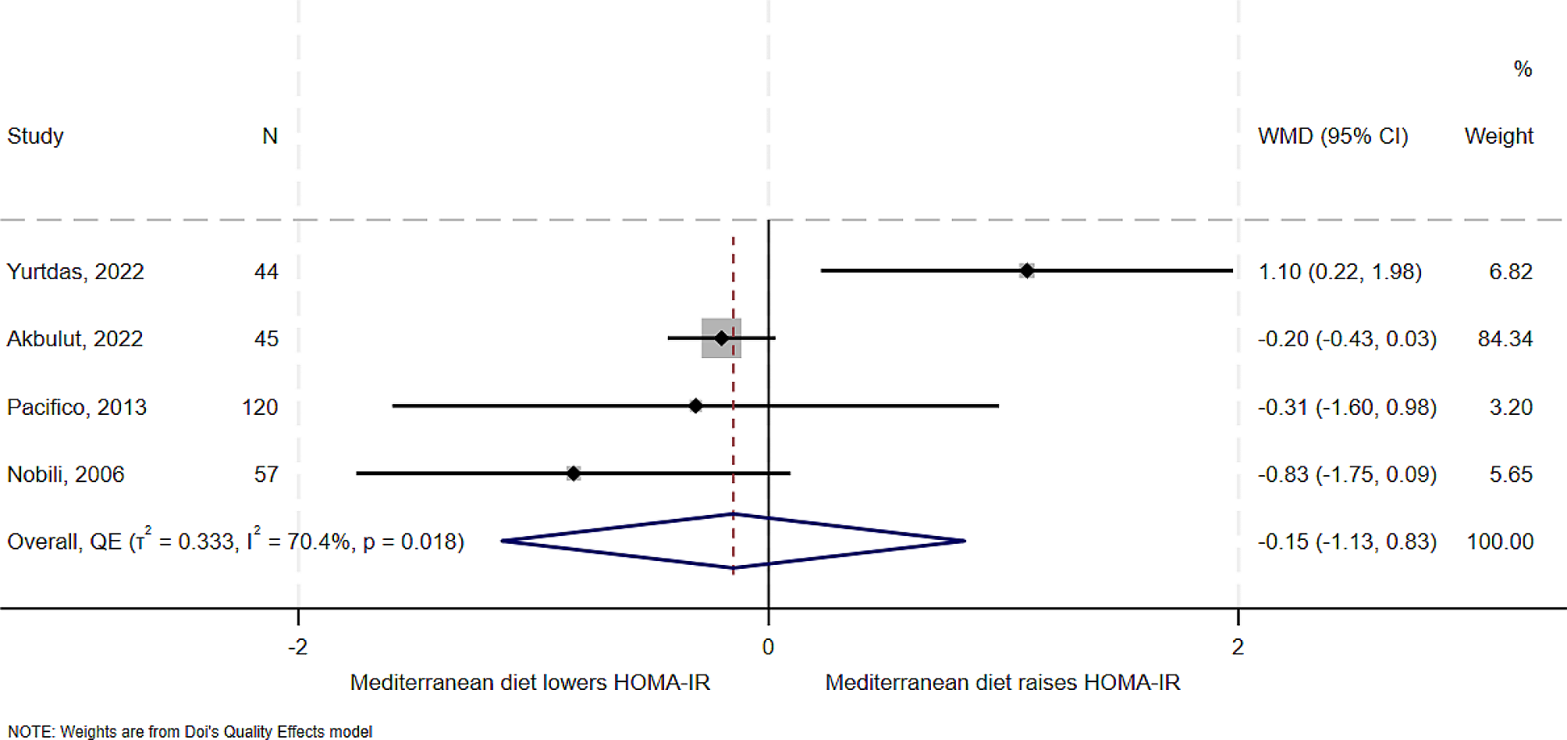
Forest plot showing effect of the Mediterranean diet compared to other diets on HOMA-IR

Four of the five studies examined the effect of Mediterranean diet on HOMA-IR. Results from these studies showed a similar effect of reduced HOMA-IR levels. One RCT [5] showed an increase in 1 unit in HOMA-IR. In overall synthesis (Fig. 5), the estimated effect of the Mediterranean diet was a mean reduction of 0.2 unit in the level of HOMA-IR (WMD − 0.15, 95% CI -1.13 to 0.83), with substantial heterogeneity (I^2^ = 70.4%, *T*^*2*^ = 0.3), with one study [5, 18] being an outlier on the Galbraith plot (Supplemental Fig. 8). In analysis without the outlying study [5, 18], the Mediterranean diet still showed benefits in reducing HOMA-IR (WMD − 0.24, 95% CI -0.46 to -0.02), with low heterogeneity and variance between studies (I^2^ = 0%, *T*^*2*^ = 0) (Supplemental Fig. 9). There was some evidence of publication bias for this outcome (Supplemental Fig. 10).

### Effect of Mediterranean diet on HDL

**Fig. 6:**
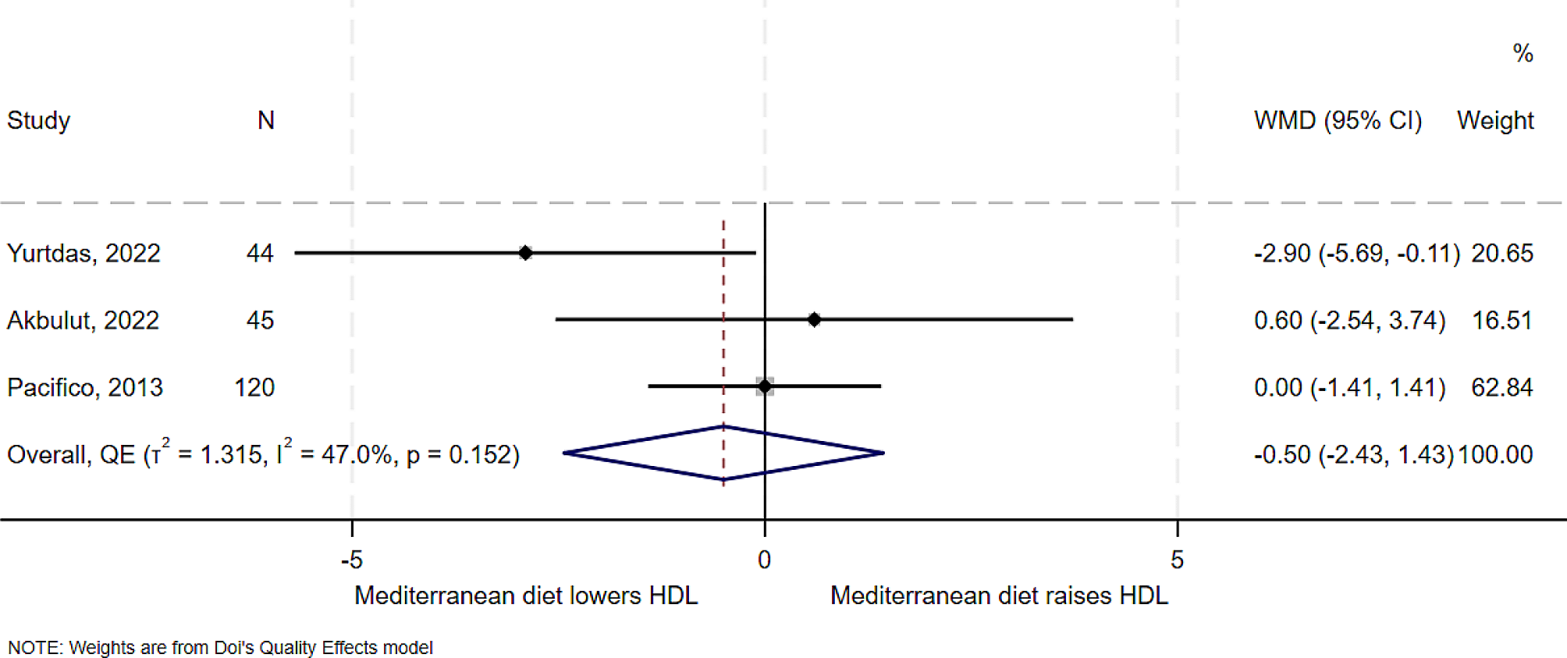
Forest plot showing effect of the Mediterranean diet compared to other diets on HDL

Three of the five studies examined the effect of Mediterranean diet on HDL. One of the trials showed an increase in HDL by 0.6 mg/dl [13] while the other [5] showed a decrease of -2.9 mg/dl. In overall synthesis (Fig. 6), the estimated effect of the Mediterranean diet was a mean reduction of 0.5 mg/dl in the level of HDL (WMD − 0.50, 95% CI -2.43 to 1.43), with low heterogeneity (I^2^ = 47%, *T*^*2*^ = 1.3). HDL had some evidence of publication bias (Supplemental Fig. 12).

### Effect of Mediterranean diet on LDL

**Fig. 7:**
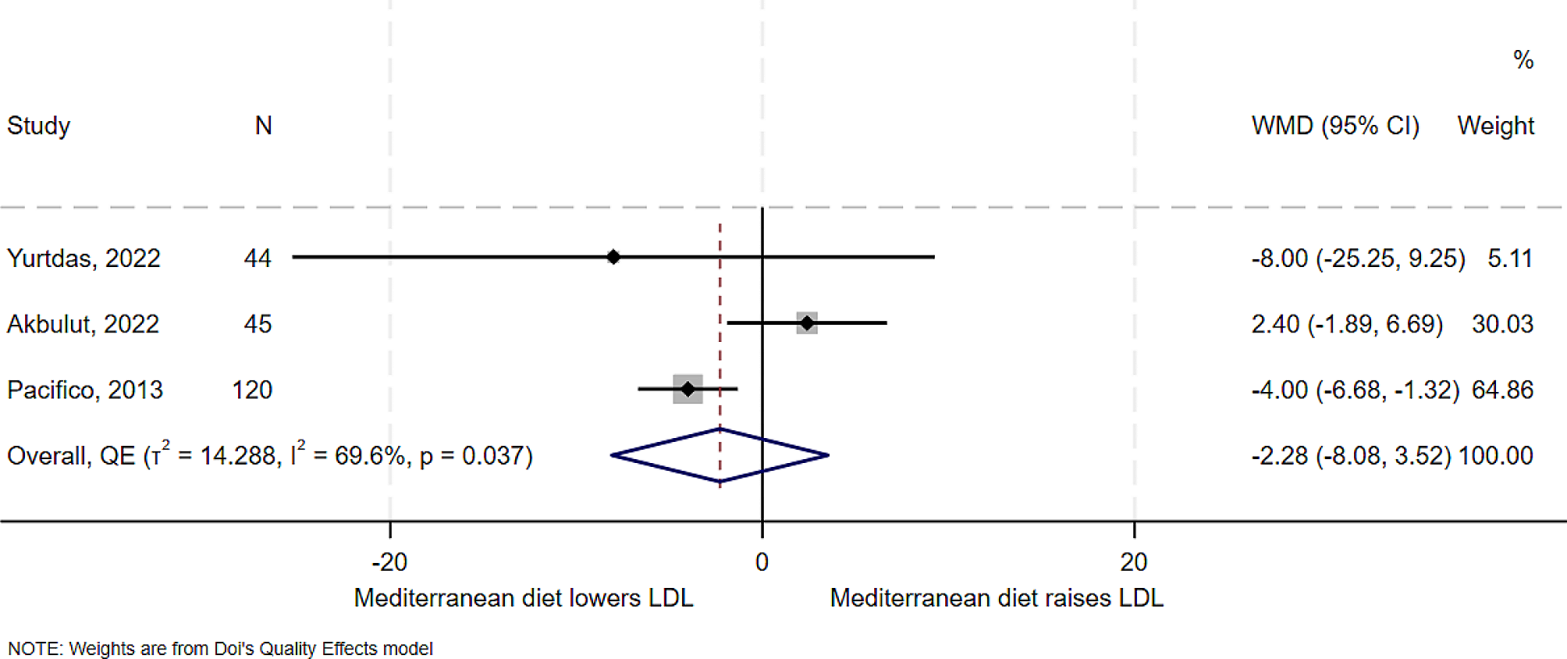
Forest plot showing effect of the Mediterranean diet compared to other diets on HDL

Three studies examined the effect of the Mediterranean diet on LDL. The studies had differing results. There was a reduction of 8 mg/dl as reported by RCT [5], while the other trial [13] showed an increase of 2.4 mg/dl. The overall effect (Fig. 7) between studies was a decrease of 2 mg/dl (WMD − 2.28, 95% CI -8.08 to 3.52). Heterogeneity and between study variance was observed to be high (I^2^ = 69.6%, *T*^*2*^ = 14.3). There was some evidence of publication bias (Supplemental Fig. 14).

### Effect of Mediterranean diet on total cholesterol

**Fig. 8:**
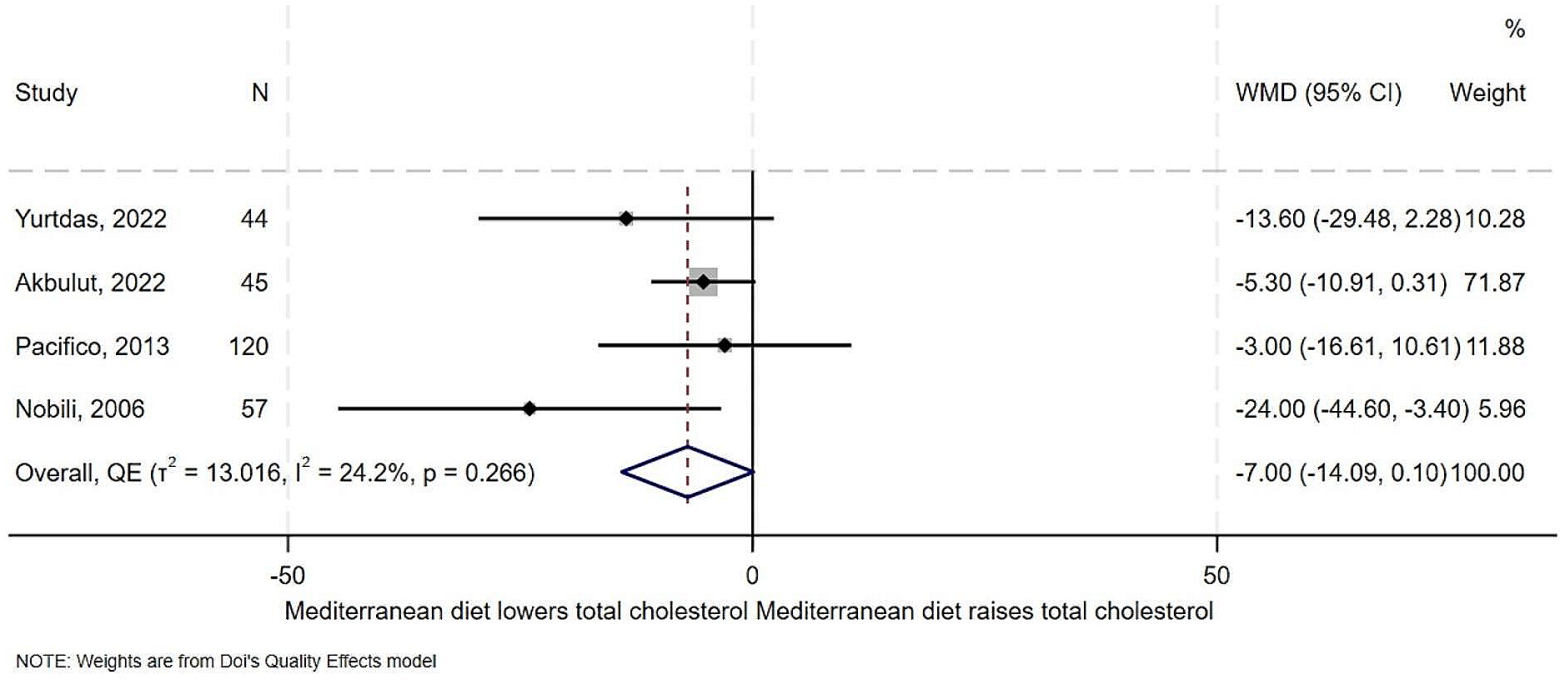
Forest plot showing effect of the Mediterranean diet compared to other diets on Total Cholesterol

Four of the five studies examined the effect of the Mediterranean diet on total cholesterol. Results from the studies showed a similar effect of reduced total cholesterol. In overall synthesis (Fig. 8), the estimated effect of the Mediterranean diet was a mean reduction of 7 mg/dl in the level of total cholesterol (WMD − 7.00, 95% CI -14.09 to 0.10), with low heterogeneity but high between study variance (I^2^ = 24.4%, *T*^*2*^ = 13.0), with no outlier on the Galbraith plot (Supplemental Fig. 15). There was some evidence of publication bias for this outcome (Supplemental Fig. 16).

### Effect of Mediterranean diet on body weight

**Fig. 9:**
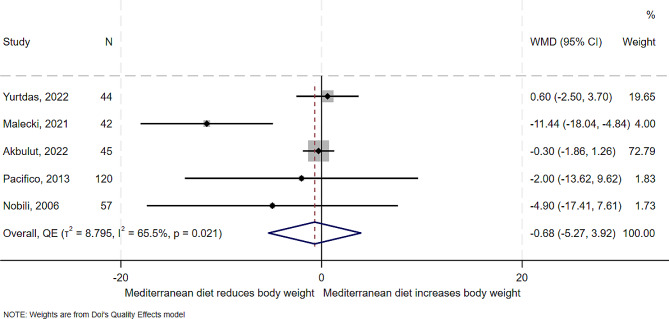
Forest plot showing the effect of the Mediterranean diet compared to other diets on body weight

All the five studies examined the effect of the Mediterranean diet on body weight. Results from the studies showed a similar effect of reduced body weight except two study that showed no reduction [5, 13]. In overall synthesis (Fig. 9), the estimated effect of the Mediterranean diet was a mean reduction of 0.7 kg in body weight (WMD − 0.68, 95% CI -5.27 to 3.92), with significant heterogeneity and high between study variance (I^2^ = 65.5%, *T*^*2*^ = 8.8). One study [20] was an outlier on the Galbraith plot (Supplemental Fig. 17). In the analysis without the outlying study [20], the Mediterranean diet showed little to no benefit in reducing body weight (WMD − 0.23, 95% CI -1.61 to 1.15), with no heterogeneity between studies (I^2^ = 0.0%, *T*^*2*^ = 0.0) (Supplemental Fig. 18). There was some evidence of publication bias for this outcome (Supplemental Fig. 19).

The summary results of all the variables of this study are shown in Table 2:

**Table 2:** Summary of the pooled analysis results

Outcome variables	Number of studies	Overall effect WMD (95% CI)	I^2^ Heterogeneity (%) / T^2^	Symmetry ( Doi plot)
ALT (U/L)	5	-10.85 (-20.03, -1.68)	42 / 38.8	Asymmetry
AST (U/L)	5	-9.26 (-17.14, -1.38)	70.7 / 42.7	Major asymmetry
GGT (U/L)	5	-1.99, (-5.09, 1.11)	25.5 / 3.1	Major asymmetry
HOMA-IR	4	-0.15 (-1.13, 0.83)	70.4 / 0.3	Major asymmetry
HDL mg/dl	3	-0.50 (-2.43, 1.43)	47 / 1.3	Major asymmetry
LDL mg/dl	3	-2.28 (-8.08, 3.52)	69.6 / 14.3	Major asymmetry
Total cholesterol mg/dl	3	-7.00 (-14.09, 0.10)	24.2 / 13.0	Major asymmetry
Body weight	5	-0.68 (-5.27, 3.92)	65.6 / 8.8	Major asymmetry

## Discussion

In this meta-analysis of two randomized controlled trials and three quasi-experimental studies, we found the Mediterranean diet was associated with modest improvements in liver function, decrease in insulin resistance, body weight and improvements in lipid metabolism, in children with MASLD. Specifically, the Mediterranean diet was associated with decreases in the inflammatory liver enzymes ALT, AST, and GGT. The Mediterranean diet did produce some small decreases in lipid metabolism, body weight and insulin resistance, but this reduction was insignificant.

In the current study, the Mediterranean diet was associated with modest improvements in liver function as shown by 11 U/L decrease in ALT and 10 U/L decrease in AST. Since the liver enzymes were elevated before the intervention, a reduction in their effect size is considered beneficial. These findings are dissimilar to the findings from a meta-analysis conducted in adults where the decrease in ALT was very small (SMD: − 0.187, 95% CI − 0.587 to 0.213) while AST and GGT were not reported [23]. Even though the results are not statistically significant, they are clinically significant. According to the guidelines [1], a decrease in ALT of 10 U/L over a period of 96 weeks gives better odds of improvement in MASLD. In the case of this review, the average duration of treatment was approximately 51 weeks. This shows that in a short duration of following the Mediterranean diet, MASLD improved substantially in children. Similar findings were documented in an adult meta-analysis [4]. GGT did not reduce likely due to the short duration of intervention in most of the included studies. The Mediterranean diet is composed of food items that provide protective effect to liver functions. It is known that polyunsaturated fatty acids consumed through diet, especially omega-3 are important in reducing the production of fat around the liver [24]. Olive oil, a major component of the Mediterranean diet, contains hydroxytyrosol shown to be beneficial in reducing oxidative stress and hepatic inflammation [24]. Another important part of the Mediterranean diet is the emphasis on the consumption of fruits and vegetables. Fruits such as tomatoes and papayas contain lycopene which is associated with reduced liver inflammation and fat accumulation [24]. All these active ingredients that work to reduce hepatic fat also help to normalize the liver enzymes and hence improve liver function. Our findings suggest the Mediterranean diet could be a low-cost public health intervention in preventing liver dysfunction in populations with a high prevalence of childhood obesity and clinically useful in treating MASLD.

The study showed reduction in insulin resistance in those following the Mediterranean diet by 0.2 unit, with substantial heterogeneity. A reduction in HOMA-IR values is considered beneficial. However, the result found in our study was not profound. The findings from a meta-analysis conducted in adults [25] on the effect of the Mediterranean diet on MASLD had different findings. They observed a significant reduction in insulin resistance (SMD: − 0.34; 95% CI: − 0.65 to − 0.03). The results from our findings could be due to the small sample size and short duration of follow up. Body weight is a significant marker for sustained improvement in liver enzymes. Our analysis showed there was only around 1 kg (WMD − 0.68, 95% CI -5.27 to 3.92) of weight loss during the duration of the study. This could be because the study duration was short for all the included studies. Or because the RCTs compared the Mediterranean diet with a low-fat, there was significant weight reduction in treatment and control groups. This explains the absence of significant differences between the treatment and control groups. An increase in HDL and, a decrease in LDL and total cholesterol is considered beneficial. In our study, there was no significant difference in the HDL and LDL levels between the two groups and negligible difference in the total cholesterol even though Mediterranean diet is known to improve LDL and HDL [21]. The two meta-analyses conducted on adults did not study the effect of Mediterranean diet on lipid metabolism [3, 25]. Our findings could be due to the short duration of studies or the comparator being low-fat diet in two of the control groups or because the participants did not have values outside the normal range before and after intervention and control. The Mediterranean diet emphasizes the consumption of wholegrain cereals which are rich in fiber. This fiber rich food helps to reduce LDL and total cholesterol. They are also low in glycemic index which helps with insulin resistance [24]. Foods high in glycemic index require insulin in large amounts, which over time can hinder the activity of insulin receptors making them less responsive and causing insulin resistance. Insulin resistance in turn causes fat oxidation in mitochondria which leads to fat accumulation in the cells [24]. To help prevent MASLD in children, public health actions need to be directed toward education about the nutrition of foods such as those high in fiber and refined carbohydrates with a high glycemic index.

The strengths of this study are that it had a robust process for the searching of studies as all possible search terms were included. The method of diagnosis of MASLD was the same in all the studies (abdominal ultrasonography) and two of the studies performed liver biopsy for the diagnosis. It is the first meta-analysis studying the effect of Mediterranean diet in children with MASLD. The outcomes were measured on the same scale for all the studies and the mean age of study participants was approximately the same across all included studies.

This meta-analysis has some limitations. There are only two RCTs examining the effect of Mediterranean diet on MASLD in children and these had low sample sizes. The intervention period between studies varied from 12 weeks to 2.5 years. A longer time for the intervention would be ideal to see the long-term impact of any dietary change. Another limitation is that we did not assess pathological markers in our analysis. This is because none of the studies included liver biopsy to assess remission of MASLD. We used proxy markers (liver enzymes) to assess improvement in MASLD. We did not include other secondary outcomes such as insulin resistance and lipid profile because there were only two RCTs and no quasi-experimental studies. The other important limitation is that three studies were non-randomized experimental studies. To take this into account, a bias-adjusted quality effects model was used in the final analysis. The heterogeneity was high for the outcomes, so a sensitivity analysis was conducted to account for this by removing outlier studies. By doing so, heterogeneity reduced significantly. Since the Mediterranean diet is a pattern of eating more than a specific diet which makes it difficult to identify which pattern was most beneficial [3, 12]. Of the five included studies, three of the studies [5, 13, 20] explicitly mentioned the intervention diet was the Mediterranean diet. However, two of the studies [18, 21] do not mention that the intervention diet was based on the Mediterranean diet. There are reasons to consider diet prescribed to the children is a low-calorie Mediterranean diet. This is because the composition of the diet including omega-6 and omega-3 in the ratio of 4:1 and emphasizing on consumption of wholegrain, fruits and vegetables which are important components of the Mediterranean diet [23]. Another reason is both studies were conducted in Italy where, since 1981 there have been campaigns to promote the Mediterranean diet [26]. The third version of the Italian dietary guidelines launched in the year 2003 has recommendations similar to the Mediterranean diet [27] such as emphasis on the consumption of extra virgin olive oil, fish, wholegrains, fruits and vegetables, nuts, and legumes [28]. The next version of the guidelines in 2018 indicates the guidelines are based on the principles of the Mediterranean diet [29]. Hence, it is possible the nutrition counseling provided to the participants was based on the Italian dietary guidelines and transitively on the Mediterranean diet. Another crucial limitation of this study is the comparison groups between studies were varied. The two trials [5, 13] compared the Mediterranean diet to low-fat diet. The three non-randomized experimental studies [18, 20, 21] compared the Mediterranean diet to an unknown baseline diet. Due to a limited number of studies, sub-group analysis based on the dietary comparators could not be performed.

## Conclusion

The results show the Mediterranean diet is better at improving liver functions compared to other diets, but different comparators are a limitation. This meta-analysis shows the Mediterranean diet improves liver function in children with MASLD. More randomized controlled trials for longer duration are needed to develop high-certainty evidence on these findings.

## Electronic supplementary material

Below is the link to the electronic supplementary material.

Supplementary Material 1

## Data Availability

The datasets used and/or analysed during the current study are openly and freely accessible from the journals that were mentioned, and all full citations are included in the reference section.

## Abbreviations

MASLD: Metabolic dysfunction-associated steatotic liver disease
MASLD: Nonalcoholic fatty liver disease
NASH: Nonalcoholic steatohepatitis
ALT: Alanine transaminase
AST: Aspartate aminotransferase
GGT: Gamma-glutamyl transferase
HOMA-IR: Homeostatic Model Assessment for Insulin Resistance
RCTs: Randomized Controlled Trials
PICO: Patient/Population, Intervention, Comparison, and Outcomes
