# Supplementary material for: Efficacy of the Mediterranean diet in treating metabolic dysfunction-associated steatotic liver disease (MASLD) in children and adolescents: a systematic review and meta-analysis
Source: BMC Public Health. 2024 Oct 3;24:2701. doi: 10.1186/s12889-024-19378-w (PMC11450996; doi:10.1186/s12889-024-19378-w)
Supplement: Supplementary file 1 — Supplementary Material 1 [file 12889_2024_19378_MOESM1_ESM.docx]

**Appendices**

**Supplemental Table 1. Risk of Bias of the Included Studies – MASTER scale**

| **Safeguard item** | Yurtdas, 2022 | Malecki, 2021 | Akbulut, 2022 | Pacifico, 2013 | Nobili, 2006 |
| --- | --- | --- | --- | --- | --- |
| 1. Data collected after the start of the study was not used to exclude participants or to select them into the analysis | 1 | 1 | 1 | 1 | 1 |
| 2. Participants in all comparison groups met the same eligibility requirements and were from the same population and timeframe | 1 | 1 | 1 | 1 | 1 |
| 3. Determination of eligibility and assignment to treatment group/ exposure strategy were synchronized | 1 | 0 | 1 | 1 | 1 |
| 4. None of the eligibility criteria were common effects of exposure and outcome | 1 | 1 | 1 | 1 | 1 |
| 5. Any attrition (or exclusions after entry) was less than 20% of total participant numbers | 1 | 1 | 1 | 1 | 0 |
| 6. Missing data was less than 20% | 1 | 1 | 1 | 1 | 1 |
| 7. Analysis accounted for missing data | 1 | 1 | 1 | 1 | 1 |
| 8. Exposure variations / treatment deviations were less than 20% | 1 | 1 | 1 | 1 | 1 |
| 9. Variations in exposure or withdrawals after start of the study were addressed by the analysis | 1 | 1 | 1 | 1 | 1 |
| 10. Procedures for data collection of covariates were reliable and the same for all participants | 1 | 1 | 1 | 1 | 1 |
| 11. The outcome was objective and/ or reliably measured | 1 | 1 | 1 | 1 | 1 |
| 12. Exposures/ interventions were objectively and/ or reliably measured | 1 | 1 | 1 | 1 | 1 |
| 13. Outcome assessor(s) were blinded | 0 | 0 | 0 | 0 | 0 |
| 14. Participants were blinded | 0 | 0 | 0 | 0 | 0 |
| 15. Caregivers were blinded | 0 | 0 | 0 | 0 | 0 |
| 16. Analyst(s) were blinded | 1 | 0 | 0 | 0 | 1 |
| 17. Care was delivered equally to all participants | 1 | 1 | 1 | 1 | 1 |
| 18. Cointerventions that could impact the outcome were comparable between groups or avoided | 1 | 1 | 1 | 1 | 1 |
| 19. Control and active interventions/ exposures were sufficiently distinct | 1 | 1 | 1 | 1 | 1 |
| 20. Exposure/intervention definition was consistently applied to all participants | 1 | 1 | 1 | 1 | 1 |
| 21. Outcome definition was consistently applied to all participants | 1 | 1 | 1 | 1 | 1 |
| 22. The time period between exposure and outcome was similar across patients and between groups or the analyses adjusted for different lengths of follow-up of patients | 1 | 1 | 1 | 1 | 1 |
| 23. Design and/or analysis strategies were in place that addressed potential confounding | 1 | 0 | 1 | 0 | 1 |
| 24. Key confounders addressed through design or analysis were not common effects of exposure and outcome | 1 | 0 | 1 | 0 | 1 |
| 25. Key baseline characteristics / prognostic indicators for the study were comparable across groups | 1 | 0 | 1 | 1 | 0 |
| 26. Participants were randomly allocated to groups with an adequate randomization process | 1 | 0 | 1 | 0 | 0 |
| 27. Allocation procedure was adequately concealed | 1 | 0 | 1 | 0 | 0 |
| 28. Conflict of interests were declared and absent | 1 | 1 | 1 | 1 | 1 |
| 29. Analytic method was justified by study design or data requirements | 1 | 1 | 1 | 1 | 1 |
| 30. Computation errors or contradictions were absent | 1 | 1 | 1 | 1 | 1 |
| 31. There was no discernible data dredging or selective reporting of the outcomes | 1 | 1 | 1 | 1 | 1 |
| 32. All subjects were selected prior to intervention/ exposure and evaluated prospectively | 1 | 1 | 1 | 1 | 1 |
| 33. Carry-over or refractory effects were avoided or considered in the design of the study or were not relevant | 1 | 1 | 1 | 1 | 1 |
| 34. The intervention/ exposure period was long enough to have influenced the study outcome | 0 | 1 | 0 | 1 | 1 |
| 35. Dose of intervention/ exposure was sufficient to influence the outcome | 1 | 1 | 1 | 1 | 1 |
| 36. Length of follow-up was not too long or too short in relation to the outcome assessment | 1 | 1 | 1 | 1 | 1 |
| **Summary count of safeguard items** | 32 | 26 | 31 | 28 | 29 |


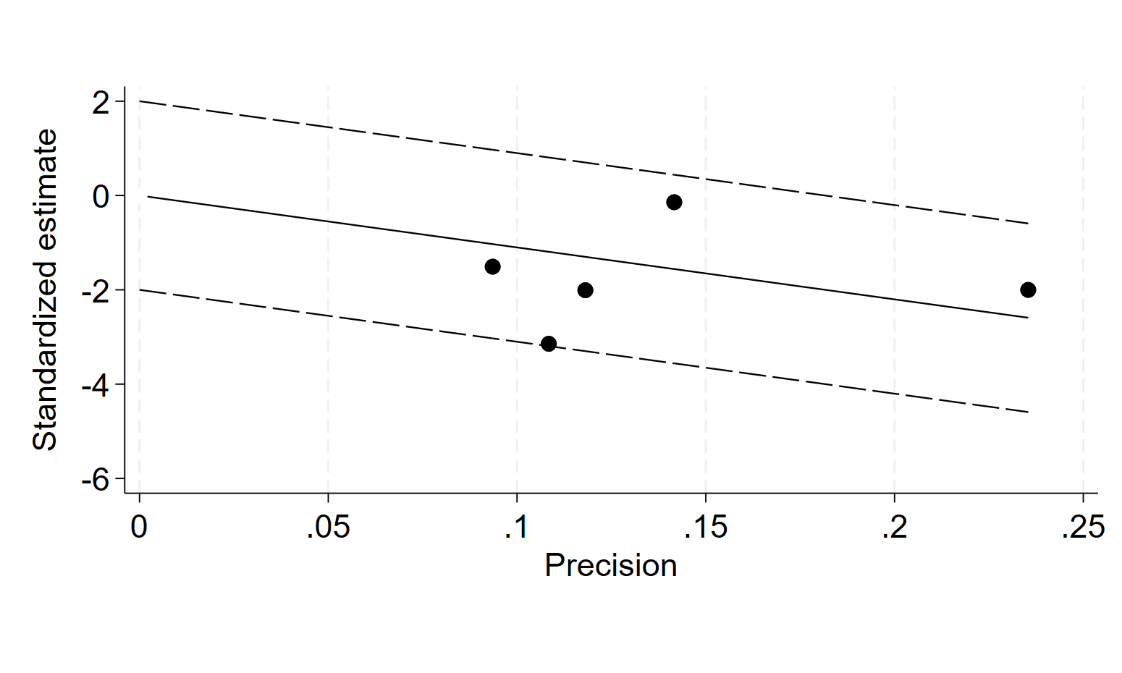


**Supplemental Figure 1: Galbraith plot of ALT**

**
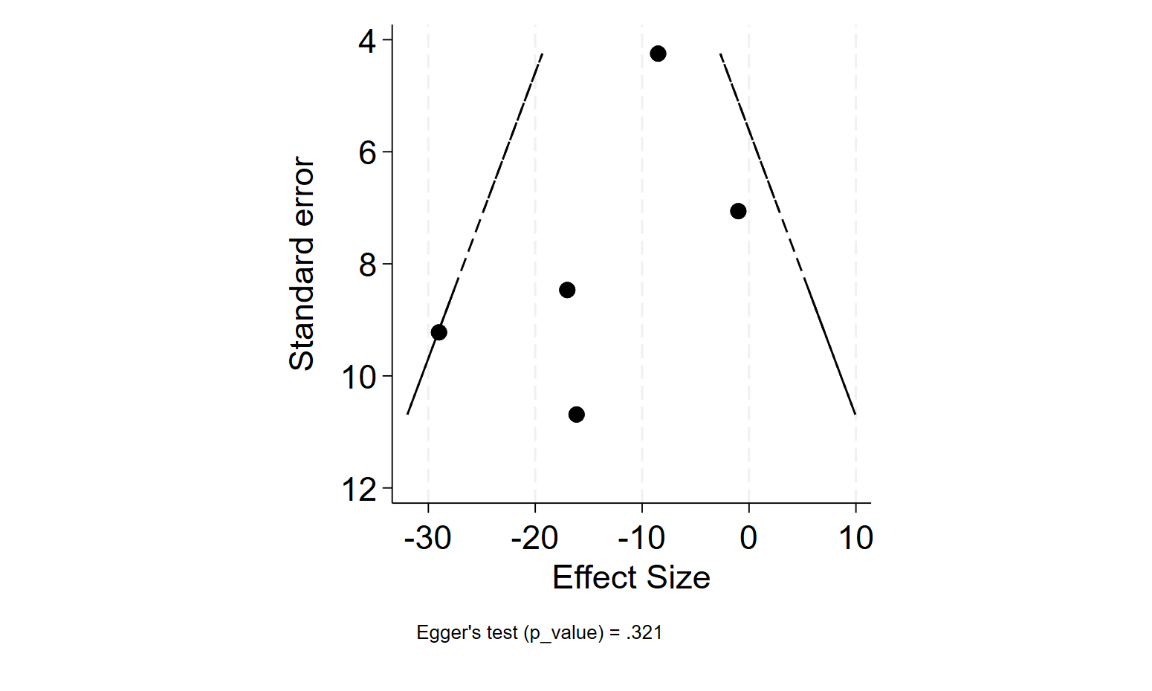

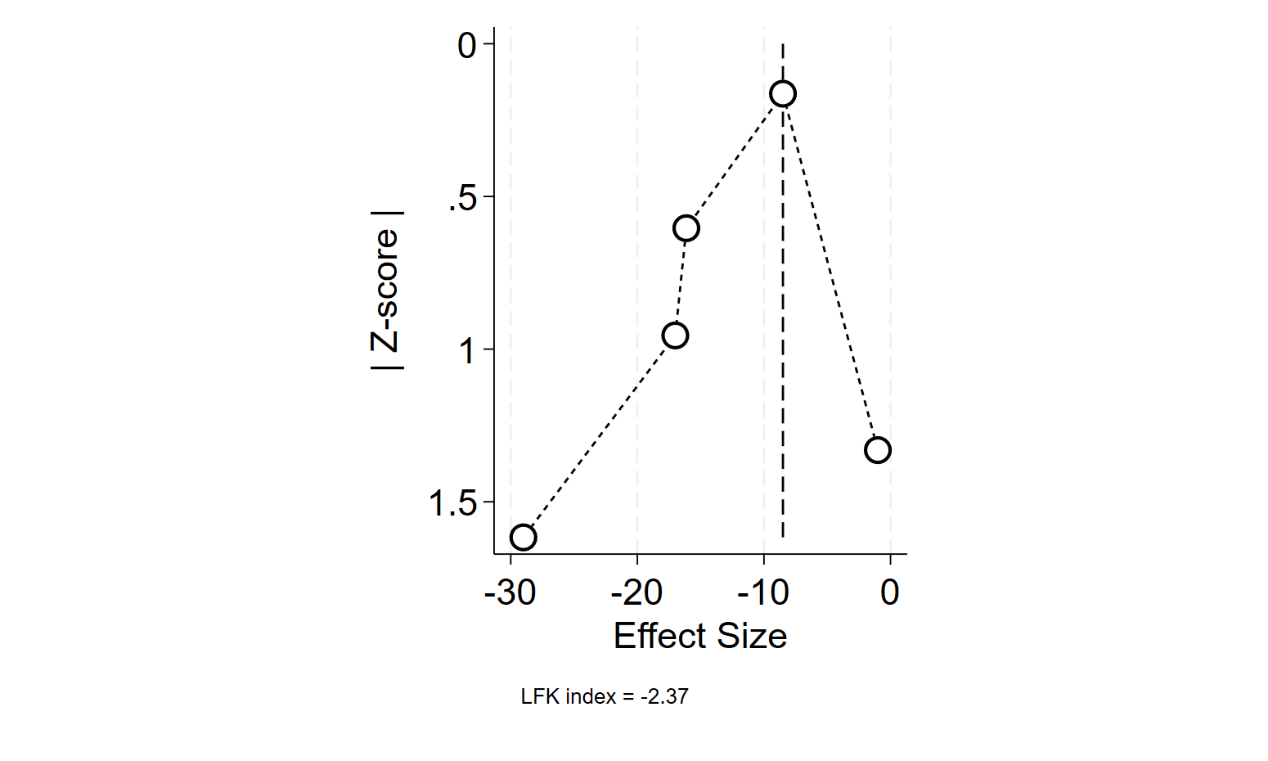
**

**Supplemental Figure 2: Assessment of publication bias in ALT**

**
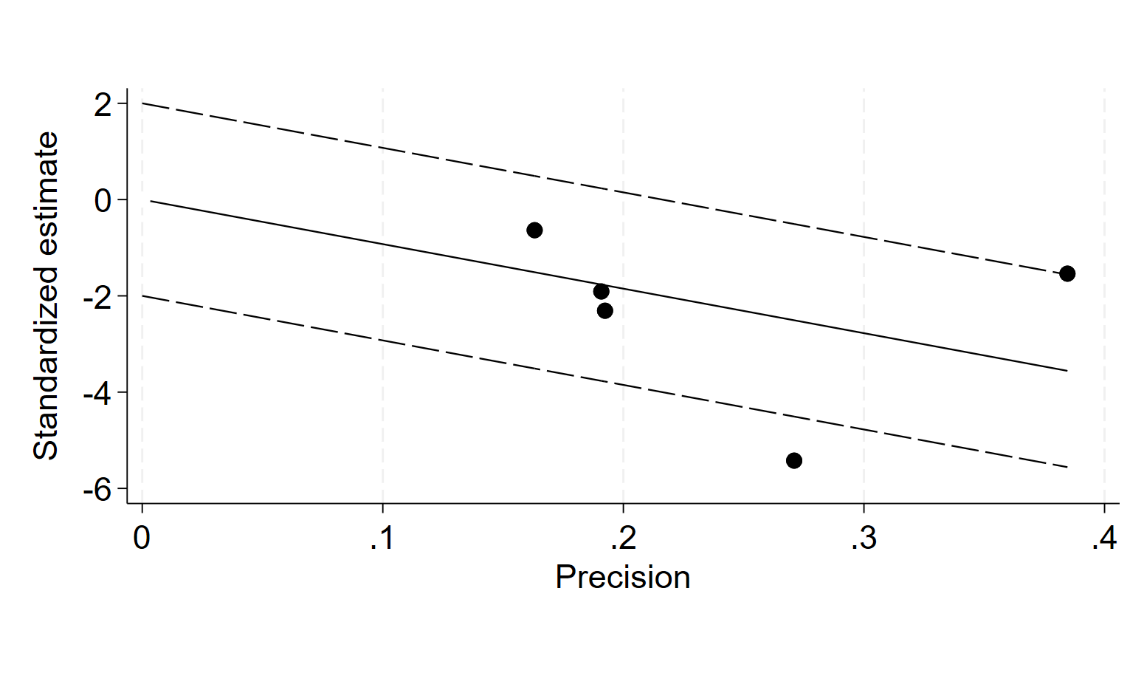
**

**Supplemental Figure 3: Galbraith plot of AST**

**
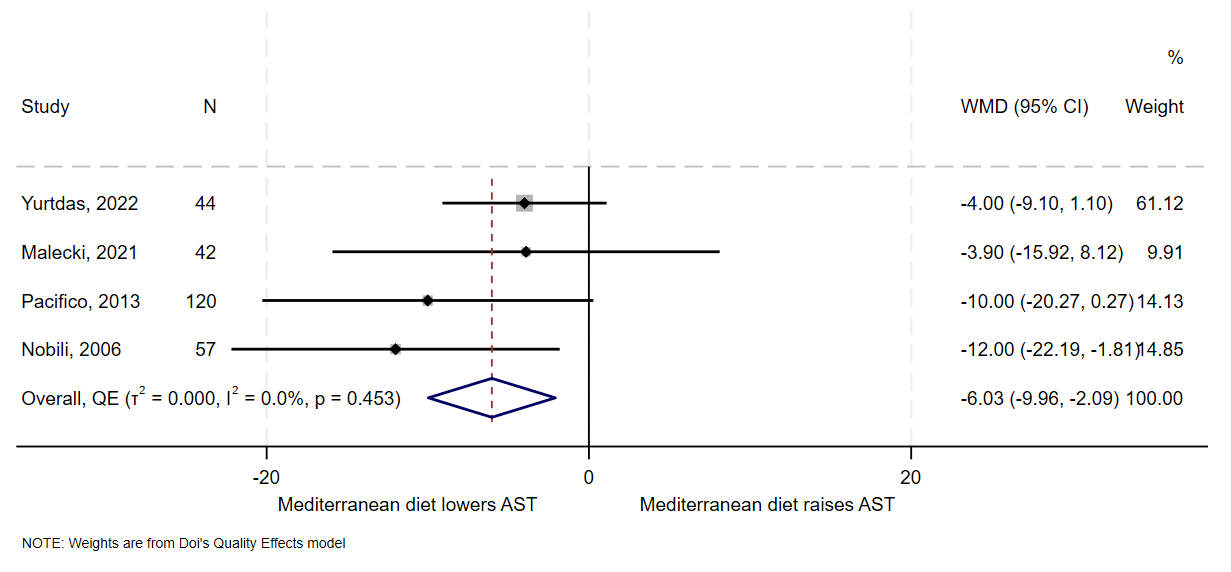
**

**Supplemental Figure 4: Forest plot after removing outlier -AST**

The outlier was (Akbulut, 2022)

**
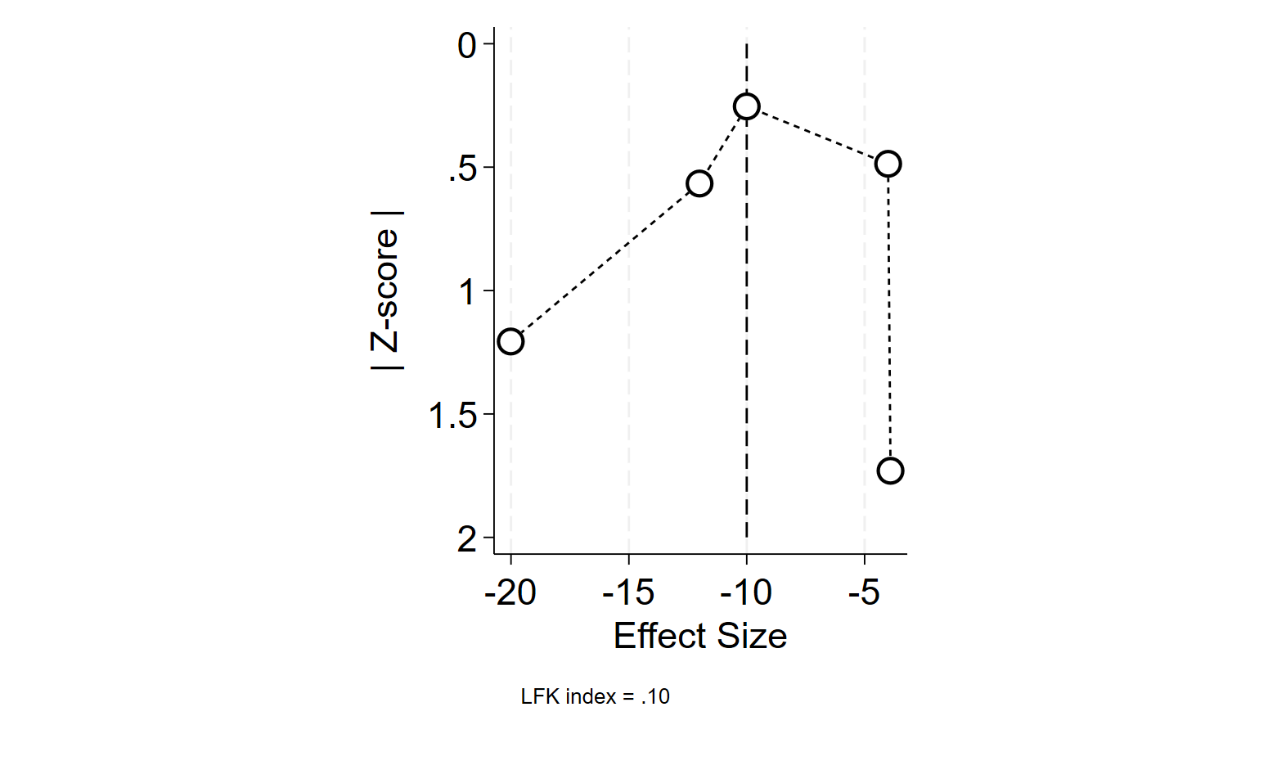

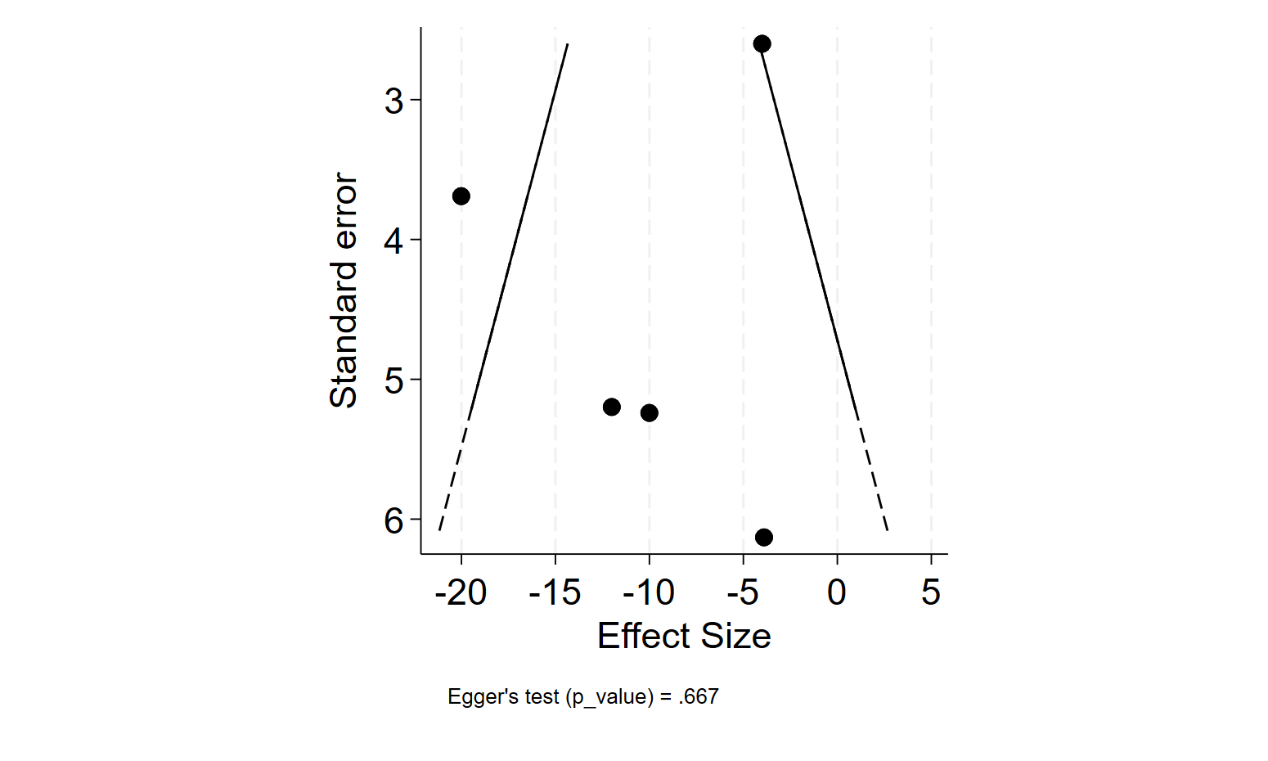
**

**Supplemental Figure 5: Assessment of publication bias in AS
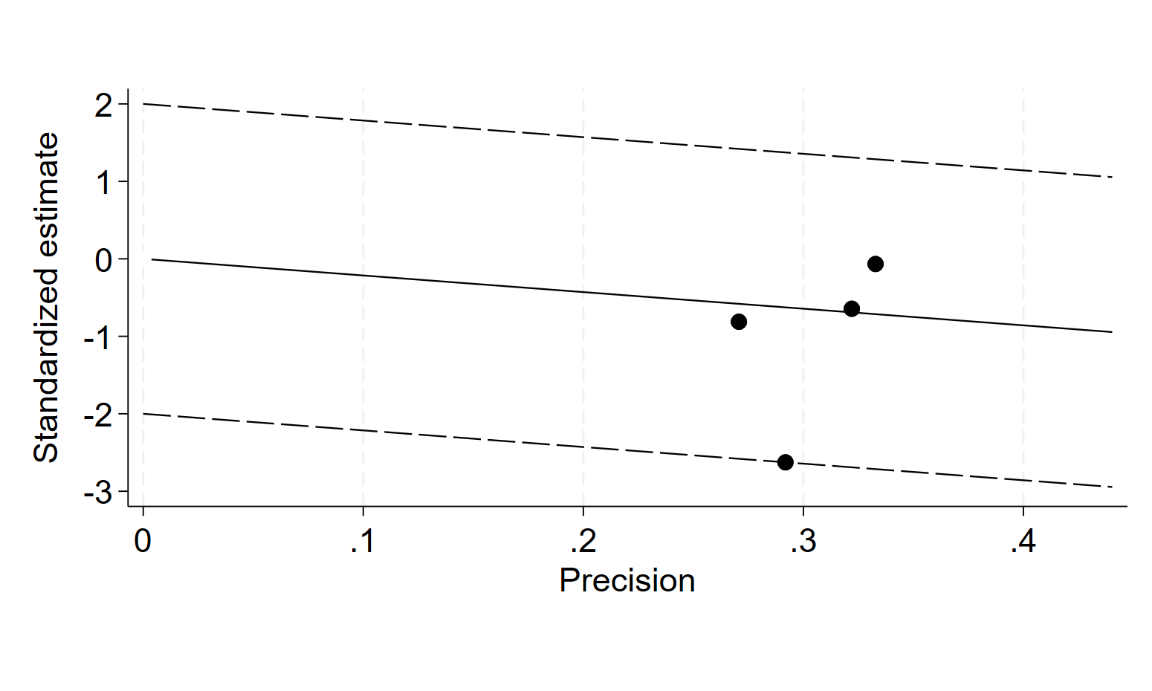
**

**Supplemental Figure 6: Galbraith plot of GGT**

**
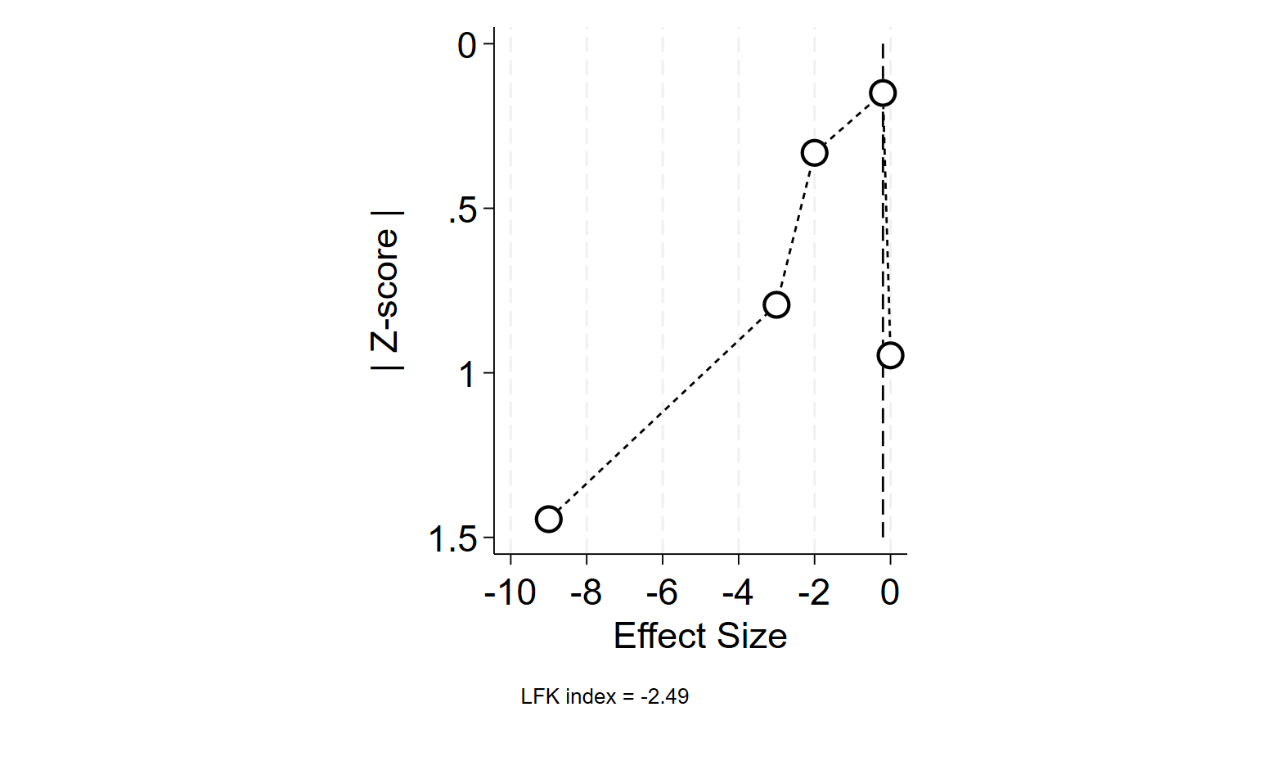

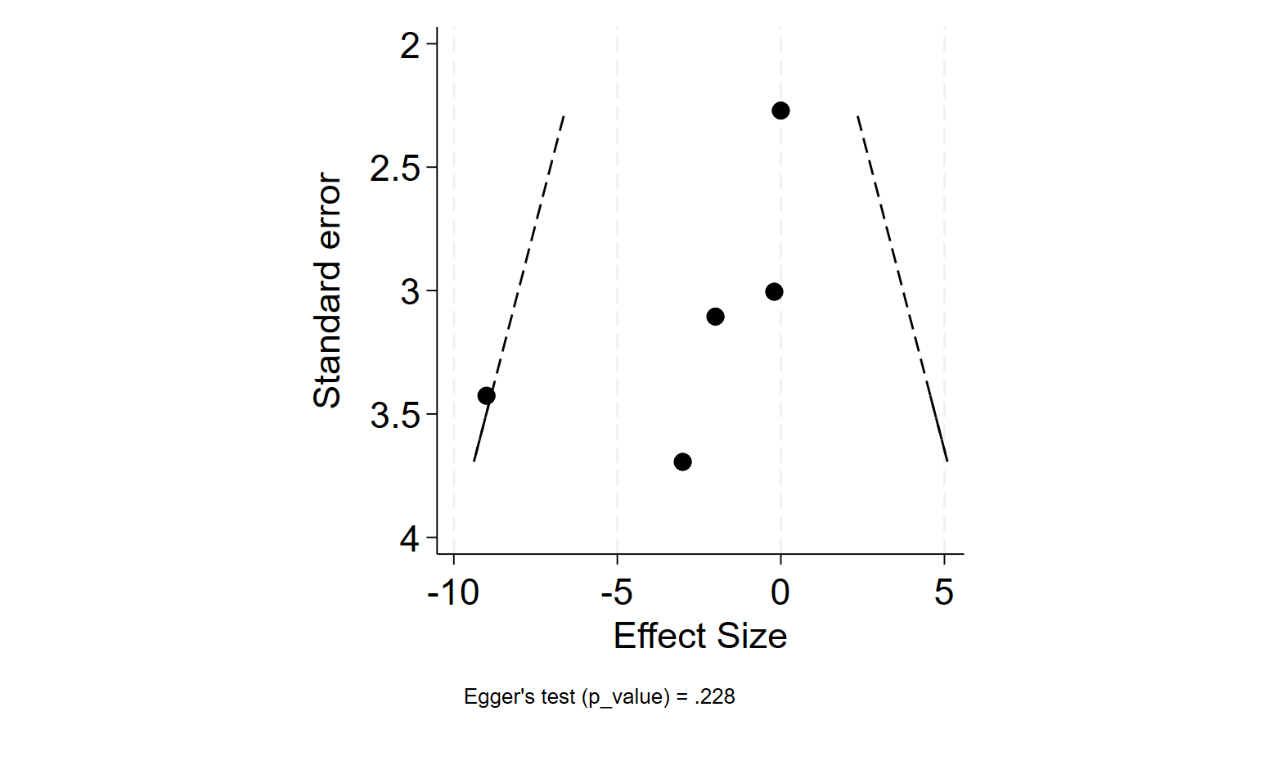
**

**Supplemental Figure 7: Assessment of publication bias in GGT**

**
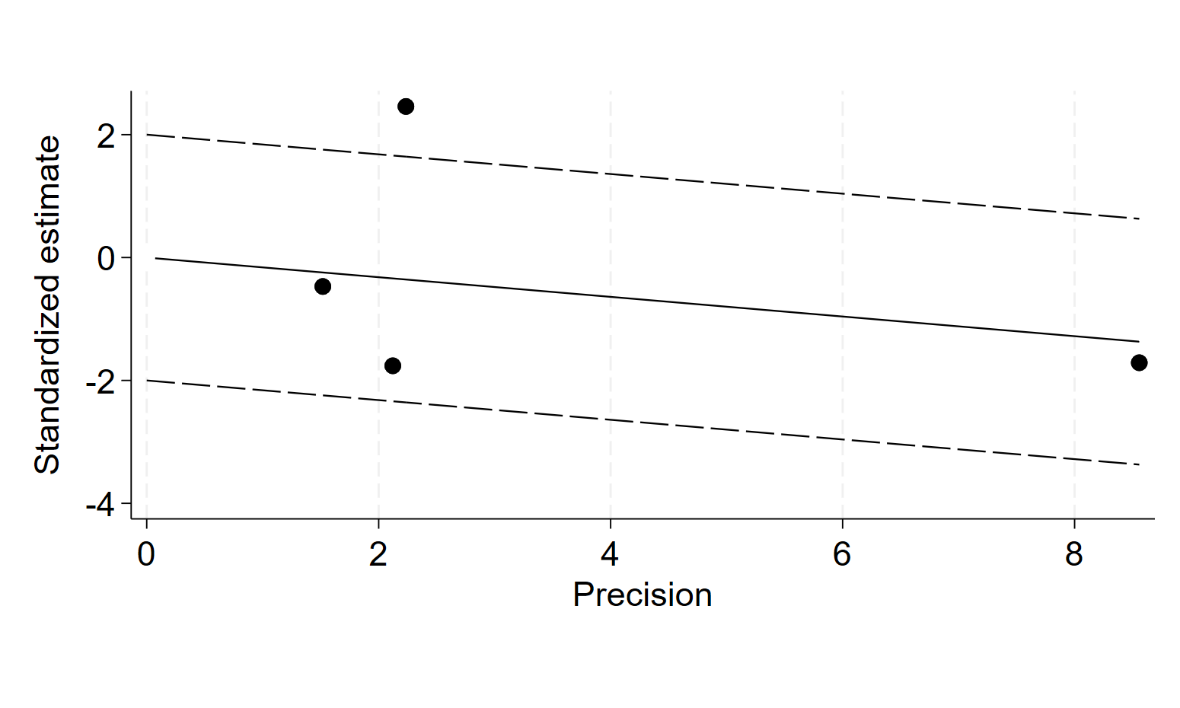
**

**Supplemental Figure 8: Galbraith plot of HOMA-IR**

**
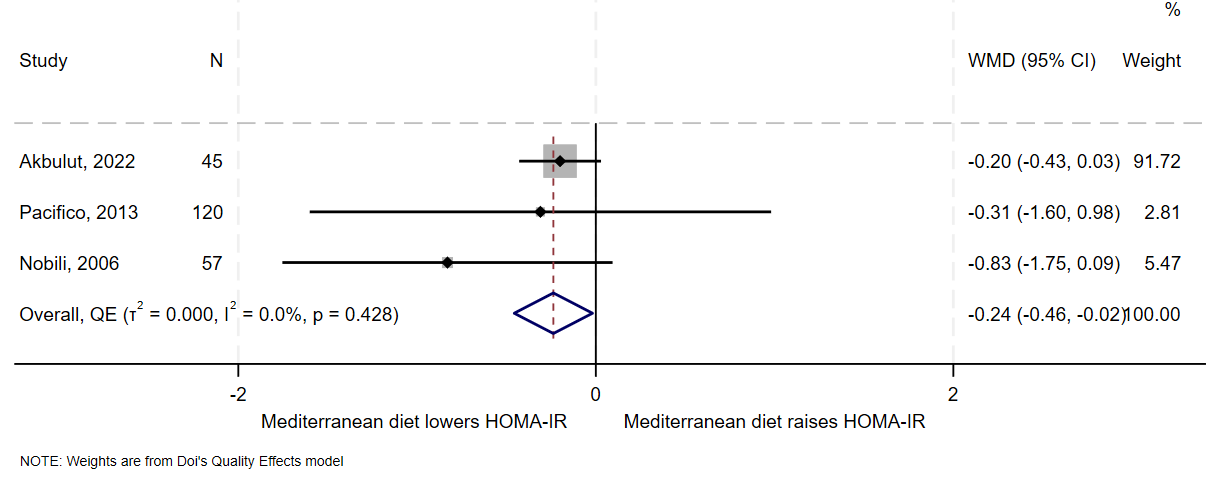
**

**Supplemental Figure 9: Forest plot after removing outlier -HOMA-IR**

The outlier was (Yurtdas, 2022)

**
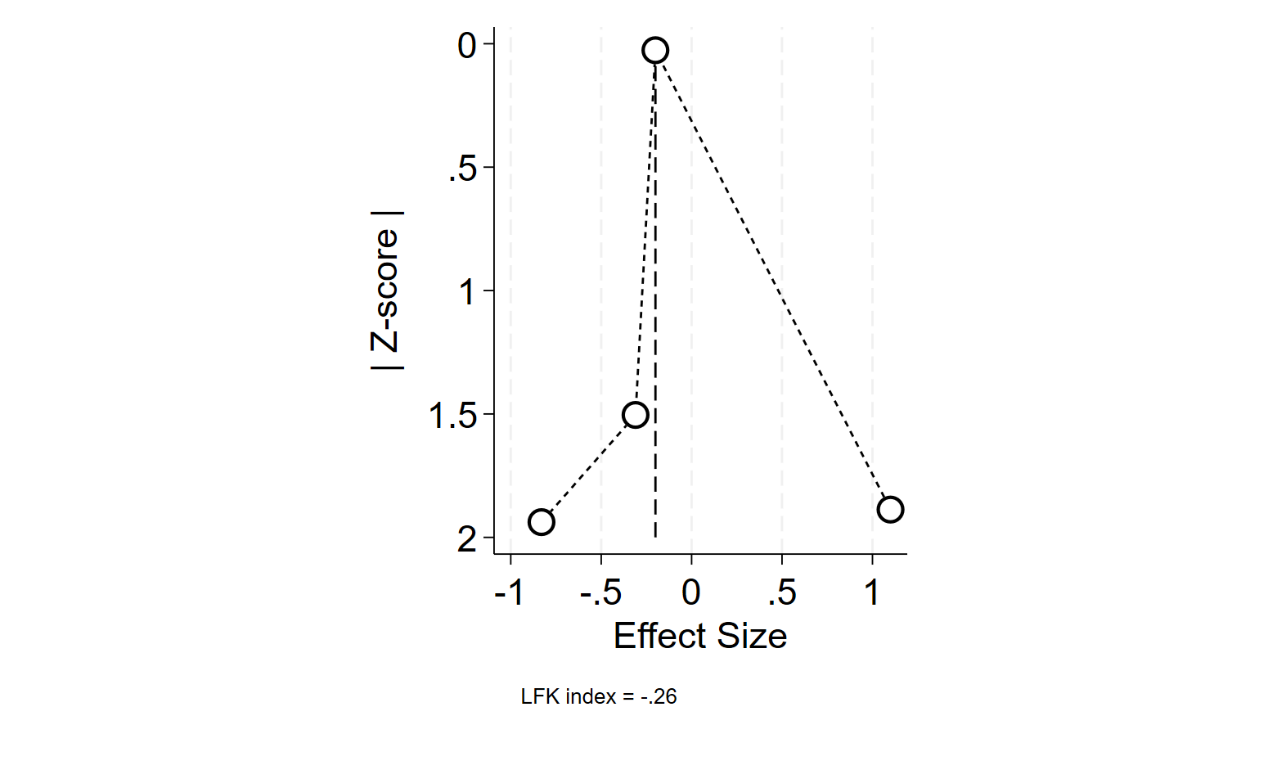

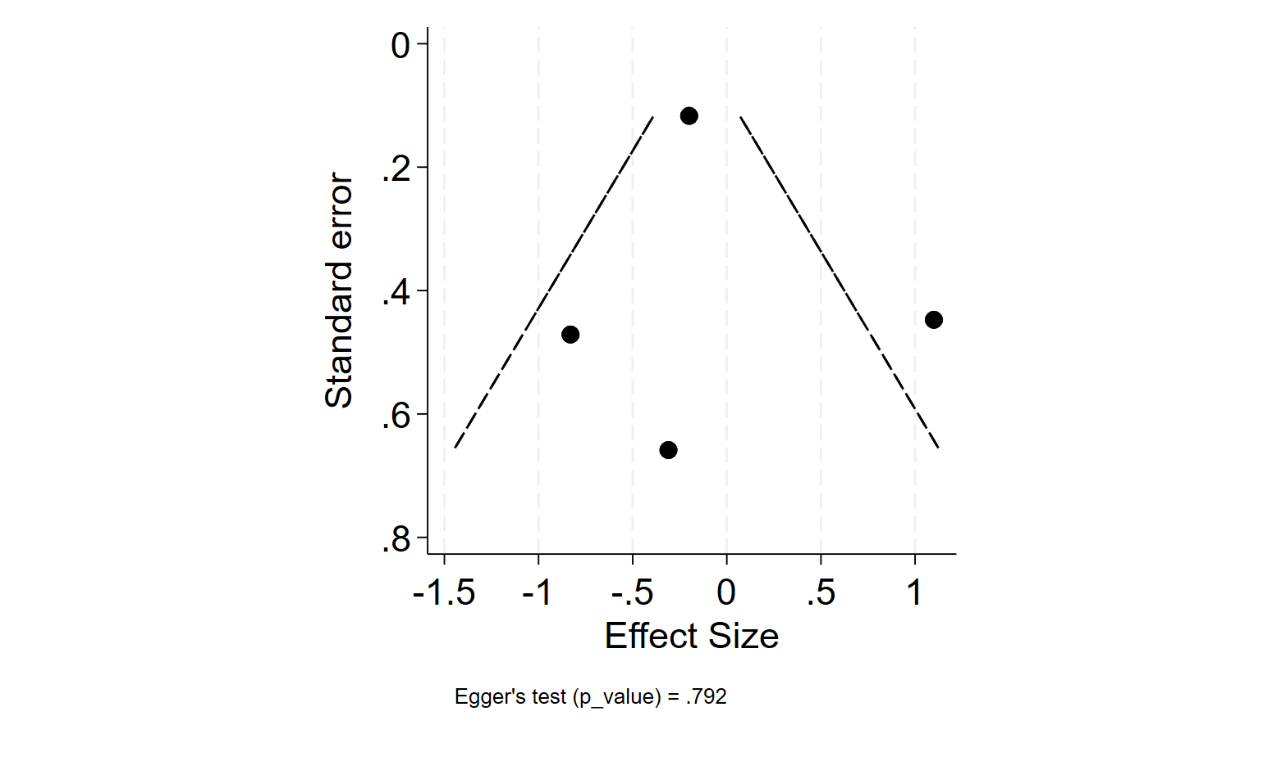
Supplemental Figure 10: Assessment of publication bias in HOMA-IR**


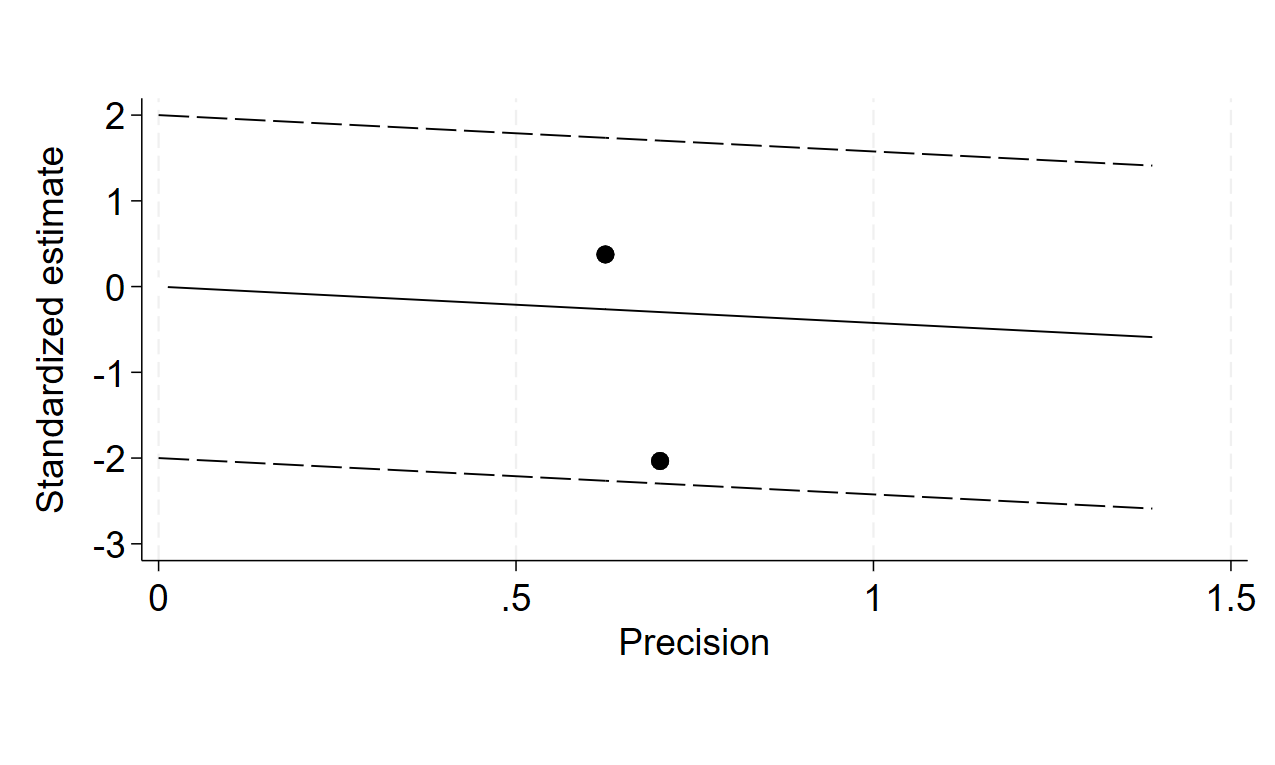


**Supplemental Figure 11: Galbraith plot of HDL**

**
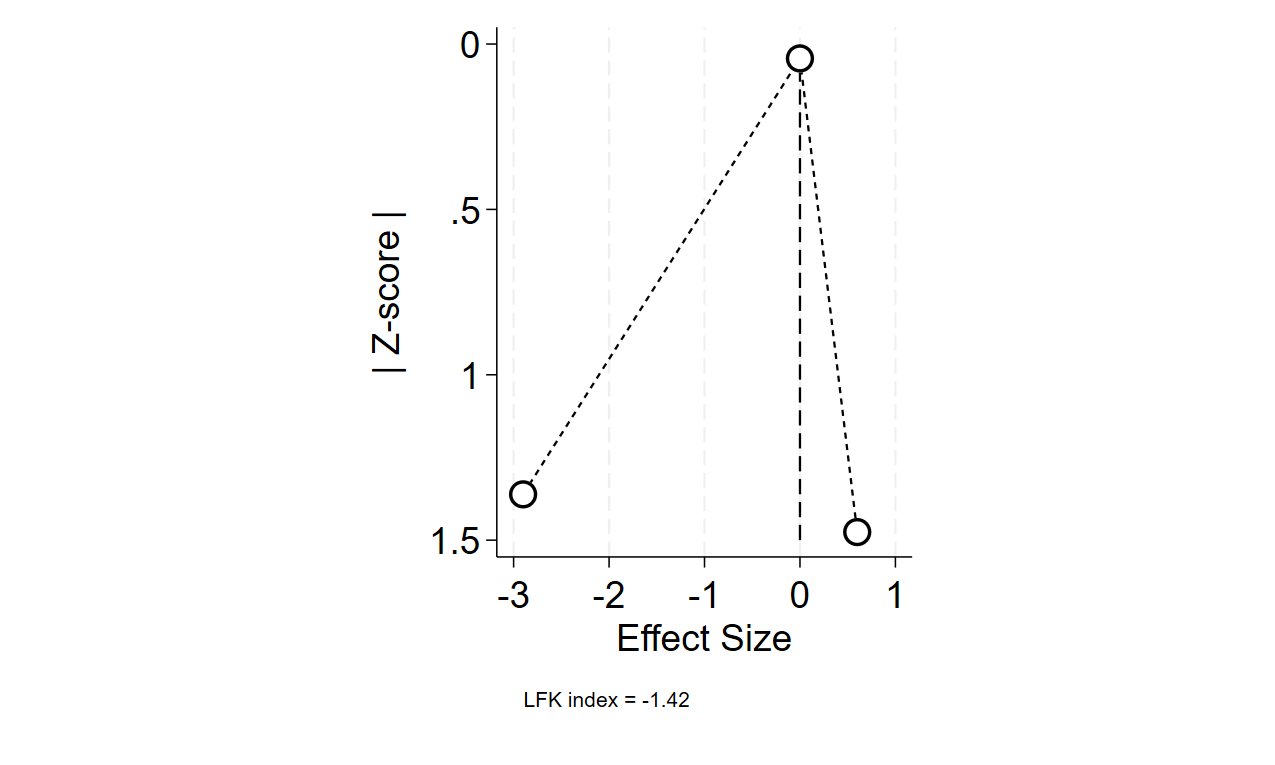

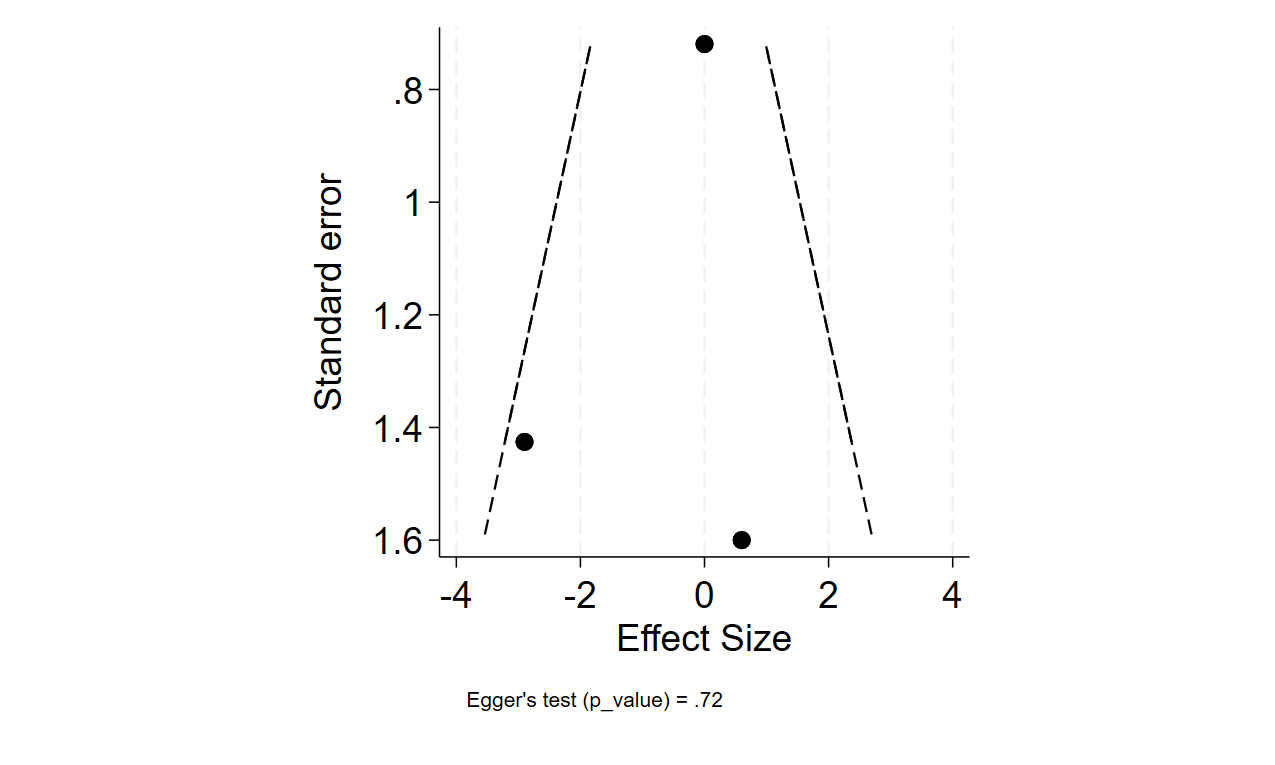
**

**Supplemental Figure 12: Assessment of publication bias in HDL**

**
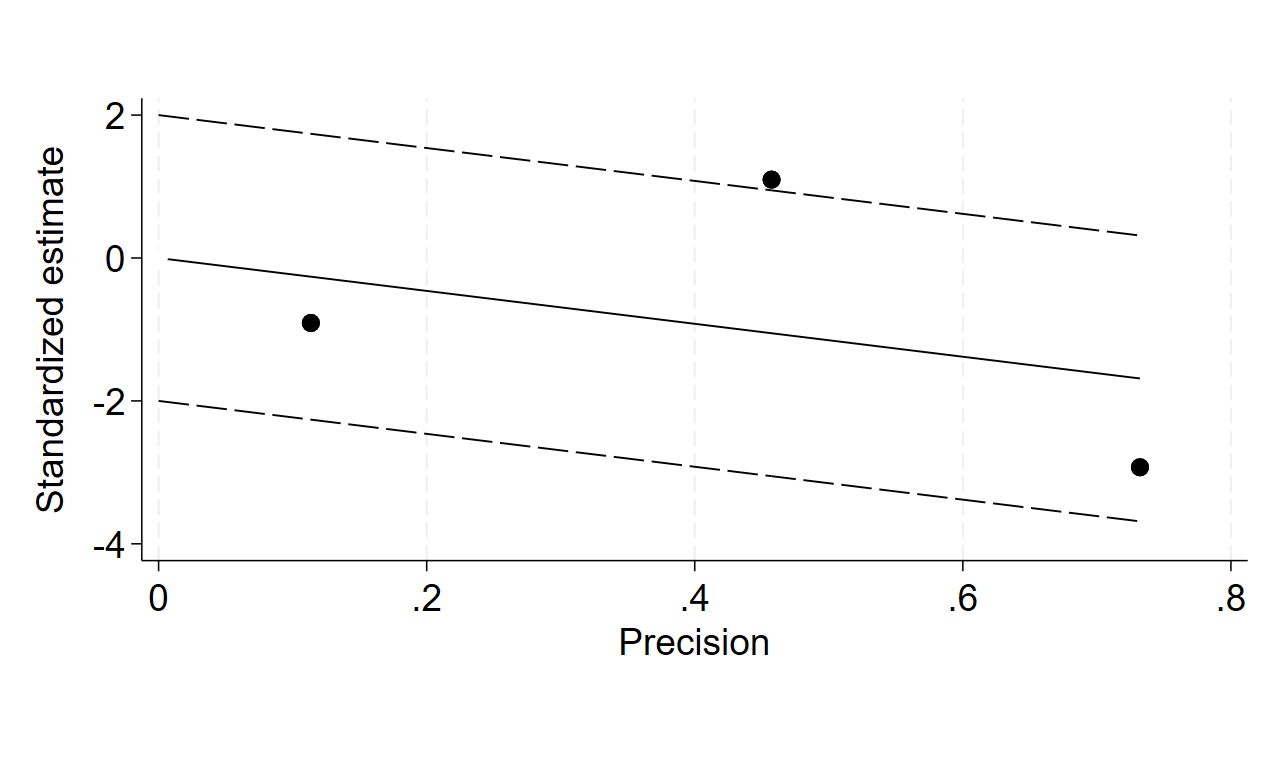
**

**Supplemental Figure 13: Galbraith plot of LDL**

**
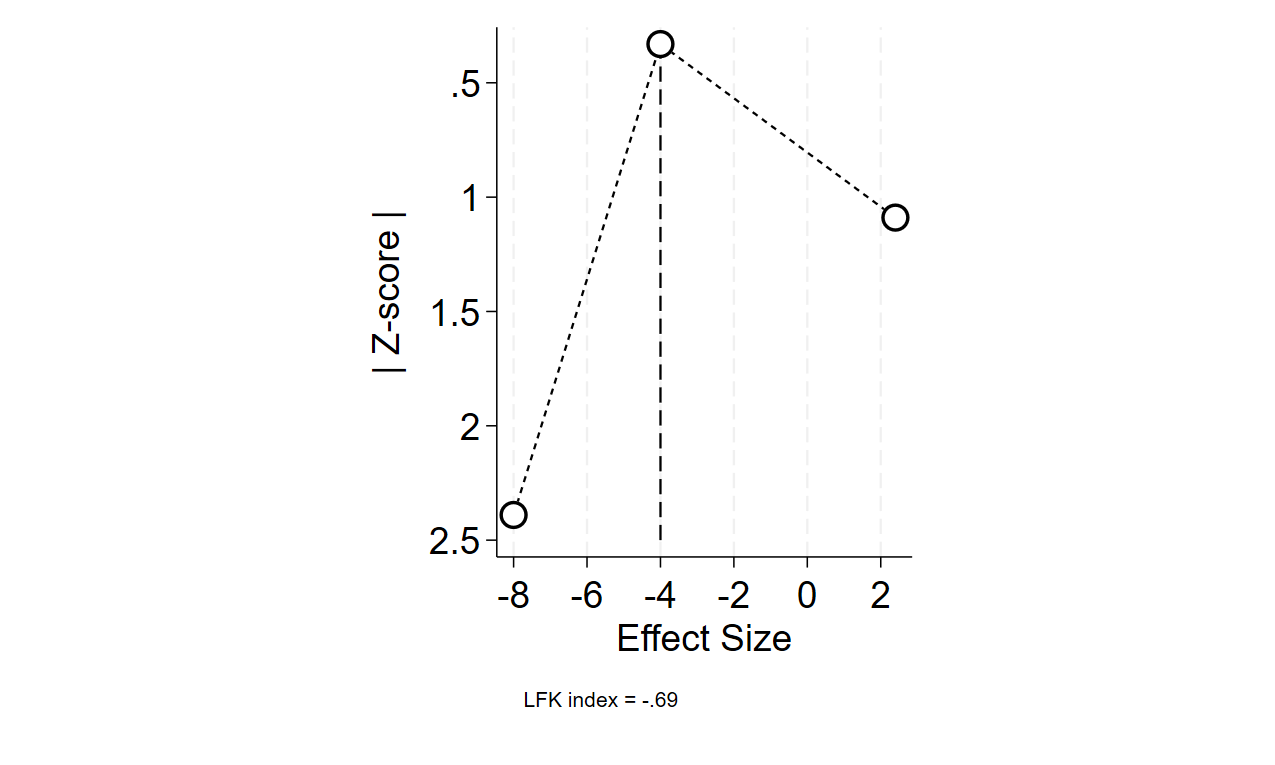

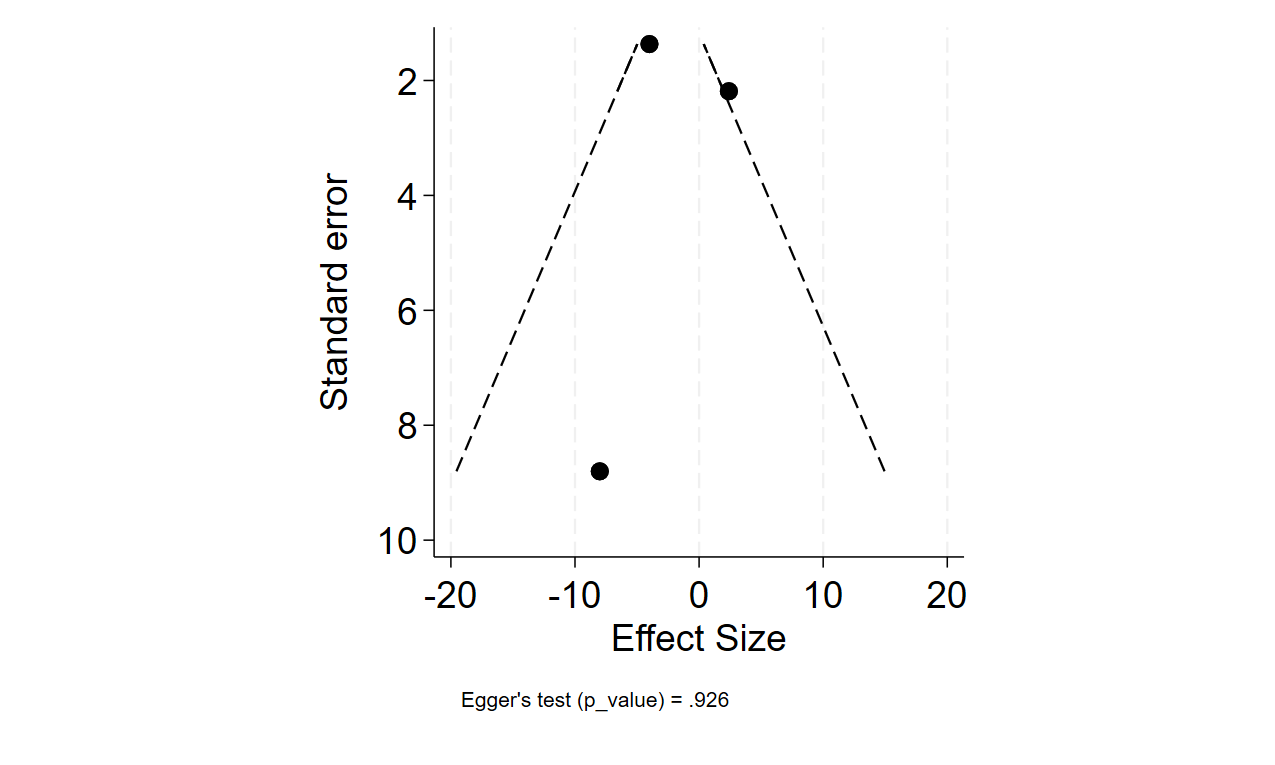
**

**Supplemental Figure 14: Assessment of publication bias in LDL**


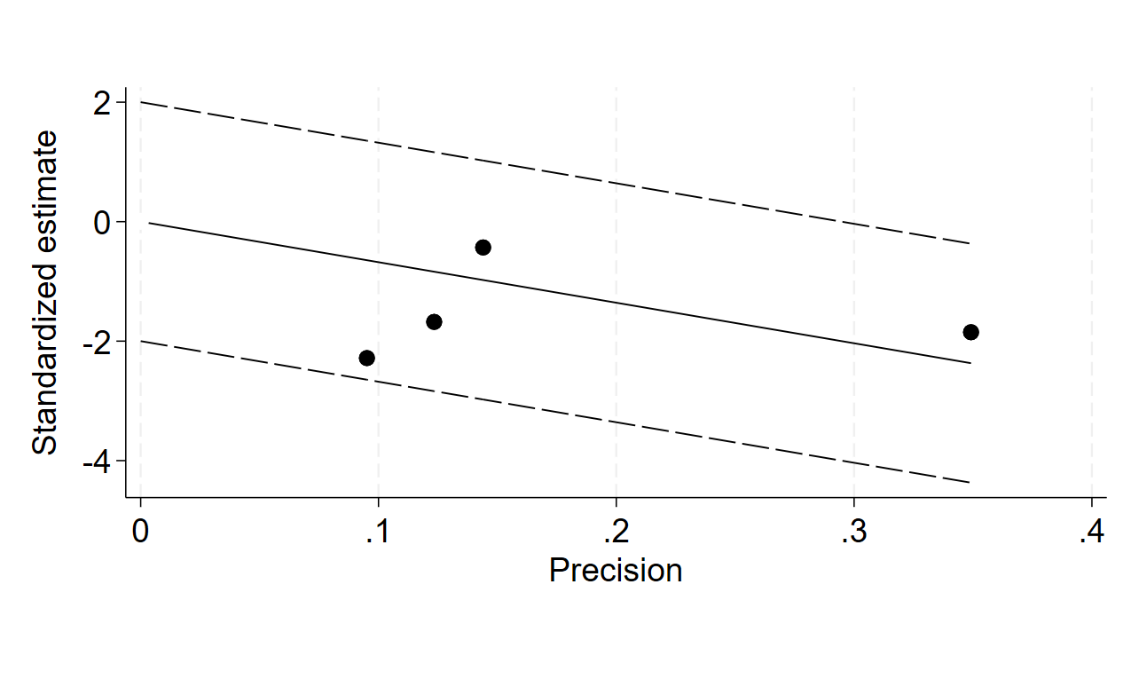
**Supplemental Figure 15: Galbraith plot of total cholesterol**


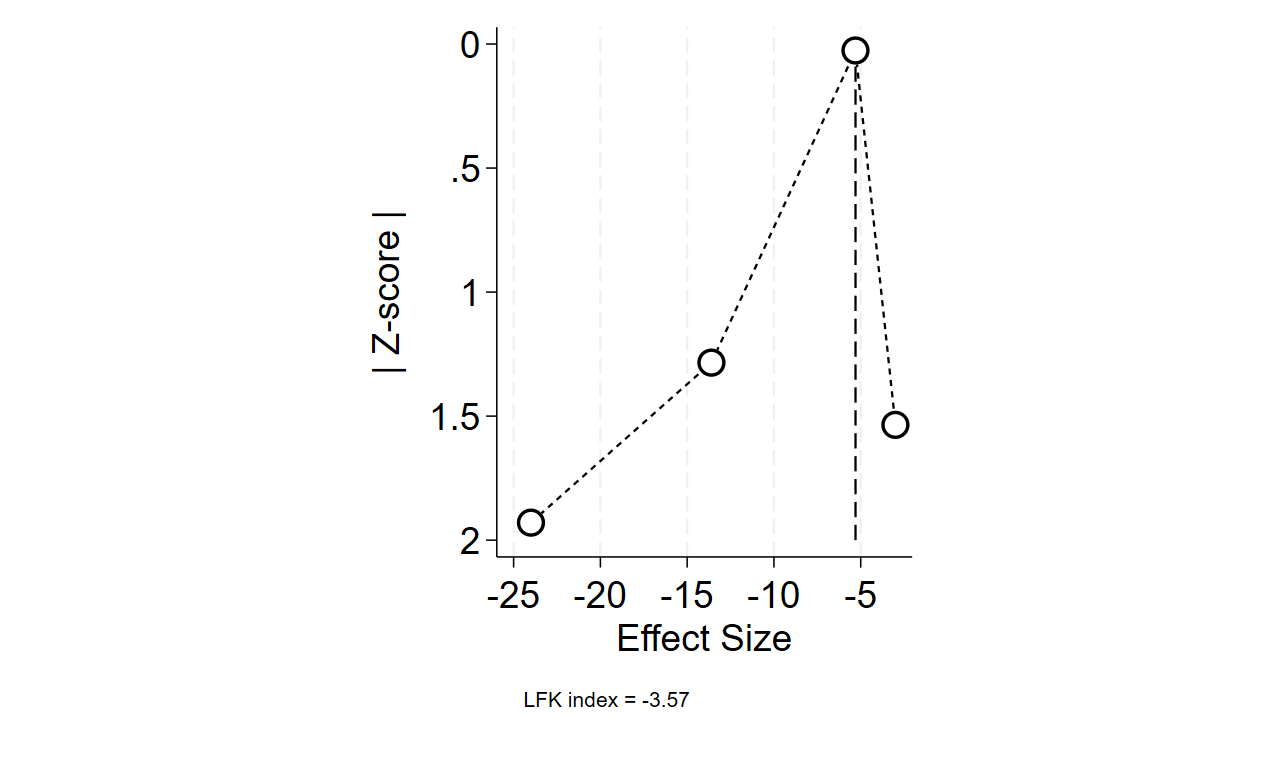

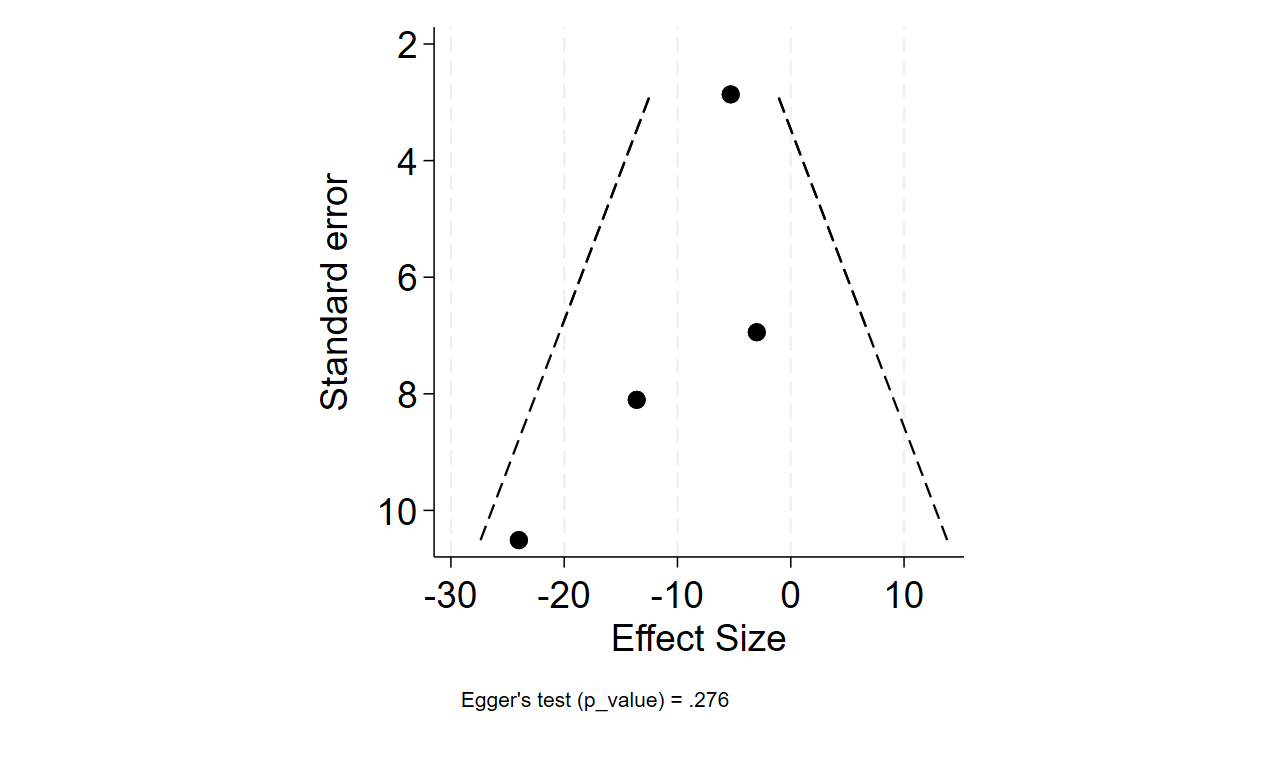


**Supplemental Figure 16: Assessment of publication bias in total cholesterol**


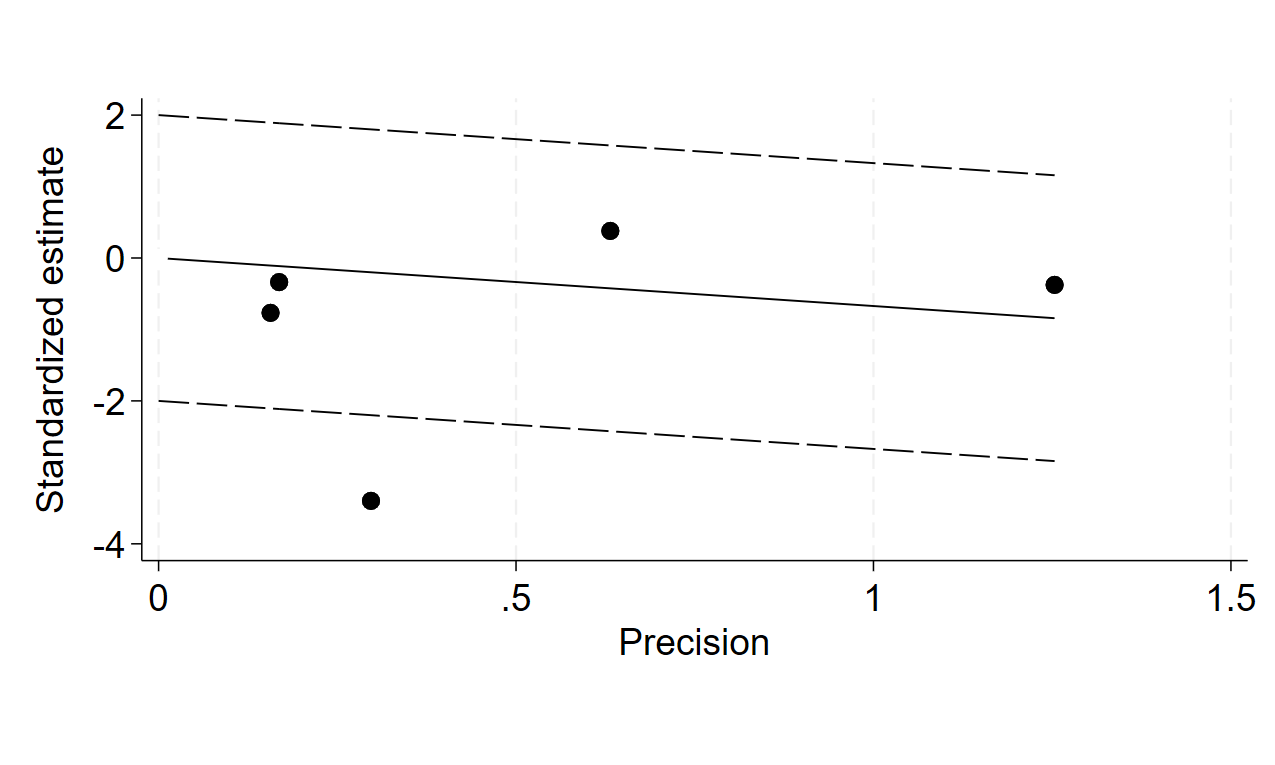


**Supplemental Figure 17: Galbraith plot of body weight**


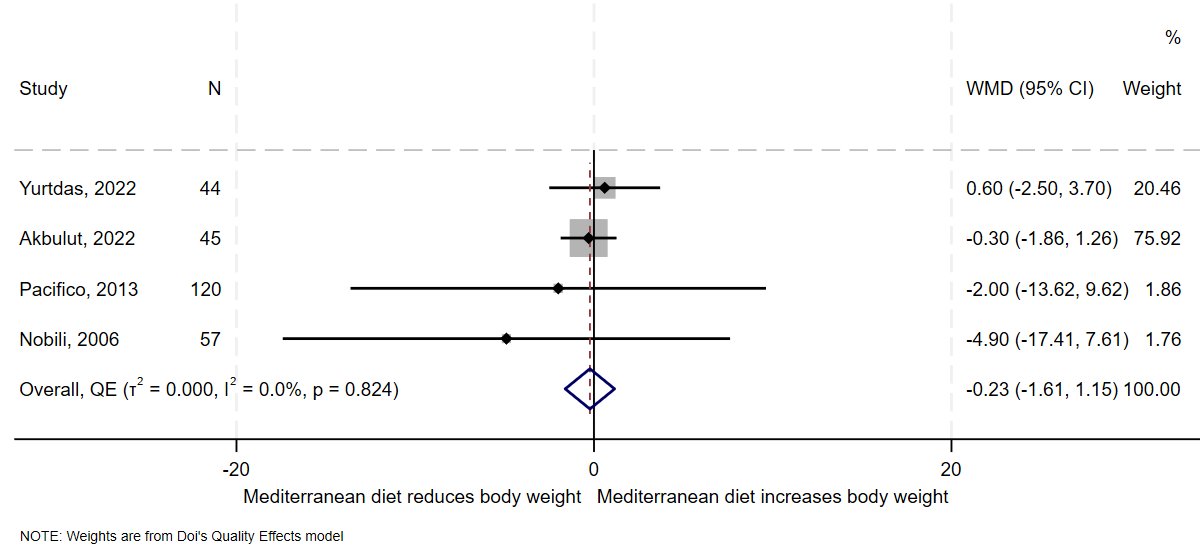


**Supplemental Figure 18: Forest plot after removing outlier -Body weight**

The outlier was (Malecki, 2021)


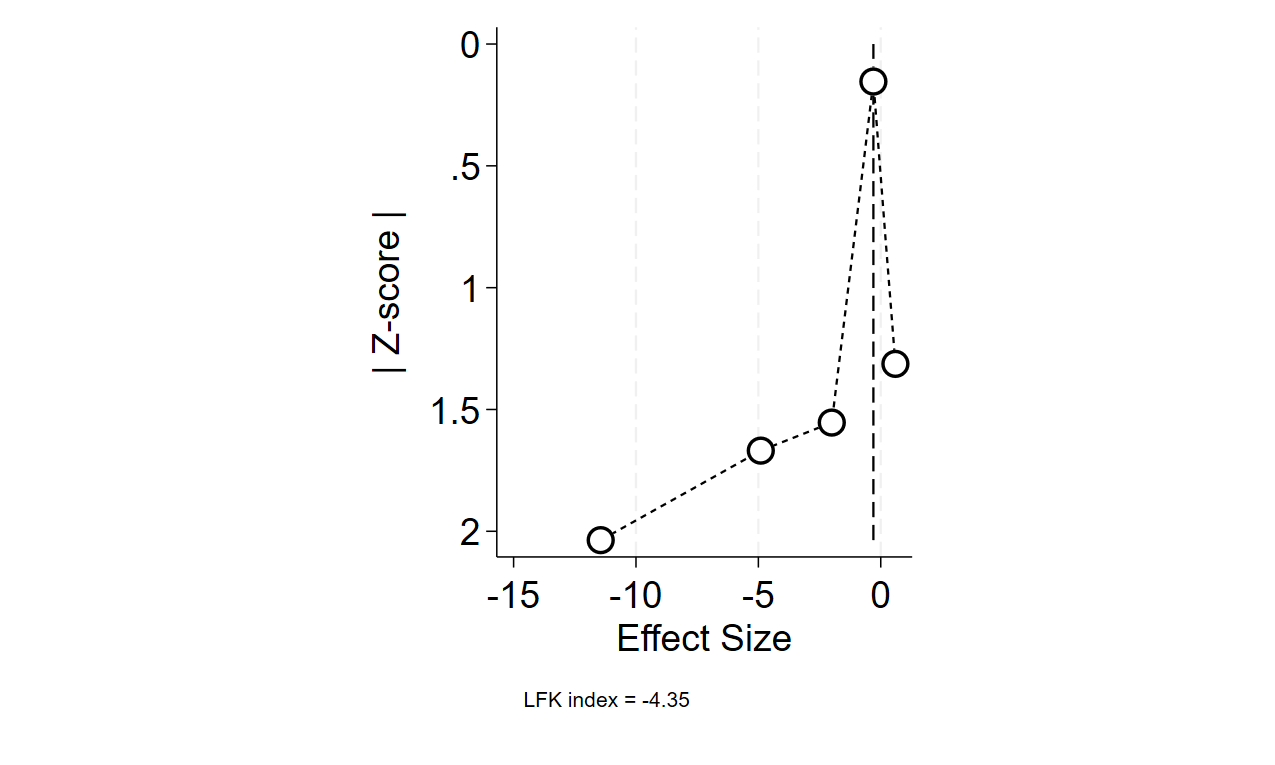

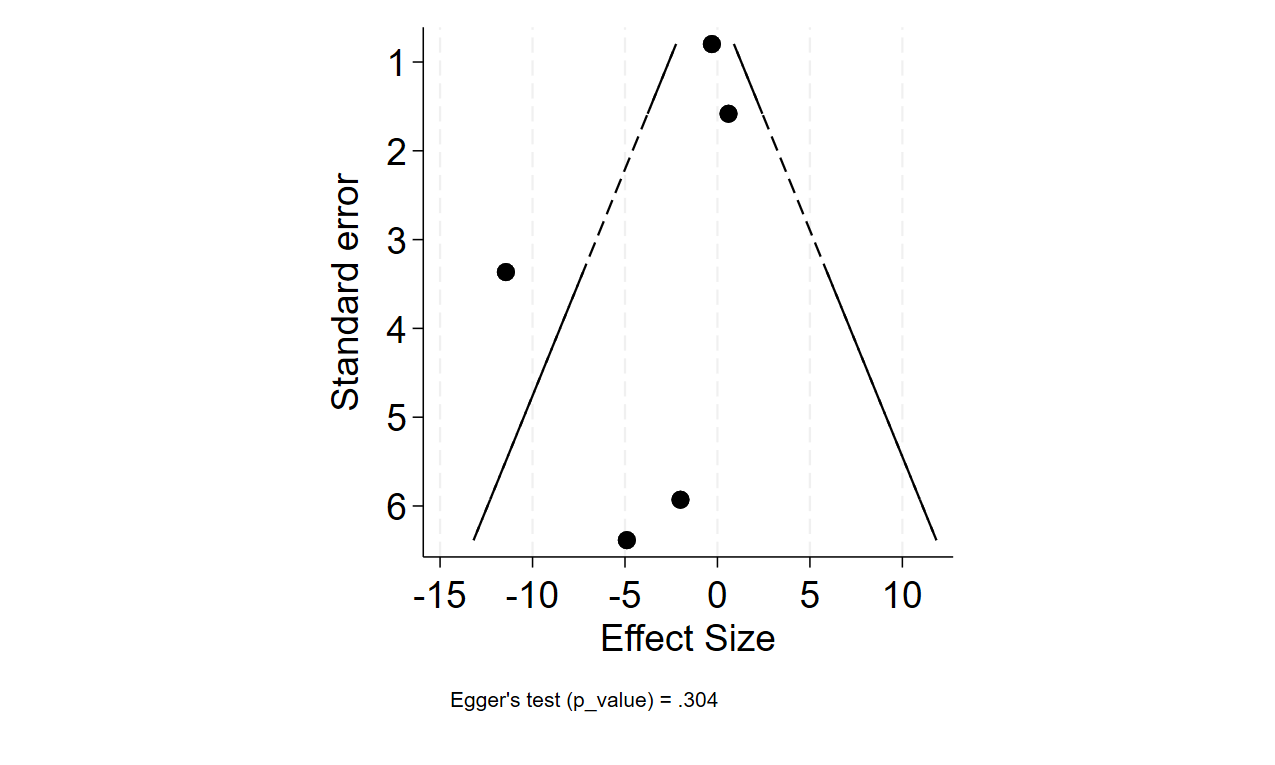


**Supplemental Figure 19: Assessment of publication bias in body weight**

**Supplemental Table 2. Search Concepts and Strategy**

##

| **Concepts** | **Terms** | |
| --- | --- | --- |
| Concept 1: NAFLD | "Non alcoholic Fatty Liver Disease” OR NAFLD* OR NASH* OR Non-alcoholic* OR Nonalcoholic* OR “Non alcoholic*” OR “Nonalcoholic Fatty Liver*” OR Fatty-liver OR “Fatty liver” OR “Nonalcoholic Steatohepatitis” OR "liver steatosis" OR liver-steatosis OR steatohepatitis* OR "hepatic steatosis" OR hepatic-steatosis | |
| Concept 2: Children and adolescents | child* OR youth OR youngster* OR preschooler* OR toddler* OR kid* OR young OR pediatric OR Adolescents OR Adolescent* OR Adolescence OR Teen* OR Teenagers OR Teenager | |
| Concept 3: MedDiet | “Mediterranean Diet” OR “Mediterranean lifestyle” OR “Mediterranean Diets” OR “cretan diet” OR Mediterranean OR “Mediterranean dietary pattern” OR “Mediterranean style diet” OR “Mediterranean diet score” OR “Mediterranean diet index” OR “Mediterranean type diet” OR MedDiet OR MeDiet OR diet OR seafood OR fruit* OR vegetable* OR “red wine” OR legumes OR “olive oil” | |
| **Database**  **Date searched** | **Search strategy** | **Result** |
| Pubmed  27/8/2023 | #1  (((((((((((((((("Non-alcoholic Fatty Liver Disease"[Mesh]) OR ("Non alcoholic Fatty Liver Disease"[Title/Abstract])) OR (NAFLD*[Title/Abstract])) OR (NASH*[Title/Abstract])) OR (Non-alcoholic*[Title/Abstract])) OR (Nonalcoholic*[Title/Abstract])) OR (Non alcoholic*[Title/Abstract])) OR ("Non alcoholic*"[Title/Abstract])) OR ("Nonalcoholic Fatty Liver*"[Title/Abstract])) OR (Fatty-liver[Title/Abstract])) OR ("Fatty liver"[Title/Abstract])) OR ("Nonalcoholic Steatohepatitis"[Title/Abstract])) OR ("liver steatosis"[Title/Abstract])) OR (liver-steatosis[Title/Abstract])) OR (steatohepatitis*[Title/Abstract])) OR ("hepatic steatosis"[Title/Abstract])) OR (hepatic-steatosis[Title/Abstract]) | 70,884 |
|  | #2  ((((((((((((((("Child"[Mesh]) AND "Adolescent"[Mesh]) OR (child*[Title/Abstract])) OR (youth[Title/Abstract])) OR (youngster*[Title/Abstract])) OR (preschooler*[Title/Abstract])) OR (toddler*[Title/Abstract])) OR (kid*[Title/Abstract])) OR (young[Title/Abstract])) OR (pediatric[Title/Abstract])) OR (Adolescents[Title/Abstract])) OR (Adolescent*[Title/Abstract])) OR (Adolescence[Title/Abstract])) OR (Teen*[Title/Abstract])) OR (Teenagers[Title/Abstract])) OR (Teenager[Title/Abstract]) | 2,482,164 |
|  | #3  (((((((((((((((("Diet, Mediterranean"[Mesh]) OR ("Mediterranean Diet"[Title/Abstract])) OR ("Mediterranean lifestyle"[Title/Abstract])) OR ("Mediterranean Diets"[Title/Abstract])) OR (Mediterranean[Title/Abstract])) OR ("Mediterranean dietary pattern"[Title/Abstract])) OR ("Mediterranean diet score"[Title/Abstract])) OR ("Mediterranean type diet"[Title/Abstract])) OR (MedDiet[Title/Abstract])) OR (MeDiet[Title/Abstract])) OR (diet[Title/Abstract])) OR (seafood[Title/Abstract])) OR (fruit*[Title/Abstract])) OR (vegetable*[Title/Abstract])) OR ("red wine"[Title/Abstract])) OR (legumes[Title/Abstract])) OR ("olive oil"[Title/Abstract]) | 628,223 |
|  | #4  (((((((((((((((((("Non-alcoholic Fatty Liver Disease"[Mesh]) OR ("Non alcoholic Fatty Liver Disease"[Title/Abstract])) OR (NAFLD*[Title/Abstract])) OR (NASH*[Title/Abstract])) OR (Non-alcoholic*[Title/Abstract])) OR (Nonalcoholic*[Title/Abstract])) OR (Non alcoholic*[Title/Abstract])) OR ("Non alcoholic*"[Title/Abstract])) OR ("Nonalcoholic Fatty Liver*"[Title/Abstract])) OR (Fatty-liver[Title/Abstract])) OR ("Fatty liver"[Title/Abstract])) OR ("Nonalcoholic Steatohepatitis"[Title/Abstract])) OR ("liver steatosis"[Title/Abstract])) OR (liver-steatosis[Title/Abstract])) OR (steatohepatitis*[Title/Abstract])) OR ("hepatic steatosis"[Title/Abstract])) OR (hepatic-steatosis[Title/Abstract])) AND ((((((((((((((((("Diet, Mediterranean"[Mesh]) OR ("Mediterranean Diet"[Title/Abstract])) OR ("Mediterranean lifestyle"[Title/Abstract])) OR ("Mediterranean Diets"[Title/Abstract])) OR (Mediterranean[Title/Abstract])) OR ("Mediterranean dietary pattern"[Title/Abstract])) OR ("Mediterranean diet score"[Title/Abstract])) OR ("Mediterranean type diet"[Title/Abstract])) OR (MedDiet[Title/Abstract])) OR (MeDiet[Title/Abstract])) OR (diet[Title/Abstract])) OR (seafood[Title/Abstract])) OR (fruit*[Title/Abstract])) OR (vegetable*[Title/Abstract])) OR ("red wine"[Title/Abstract])) OR (legumes[Title/Abstract])) OR ("olive oil"[Title/Abstract]))) AND (((((((((((((((("Child"[Mesh]) AND "Adolescent"[Mesh]) OR (child*[Title/Abstract])) OR (youth[Title/Abstract])) OR (youngster*[Title/Abstract])) OR (preschooler*[Title/Abstract])) OR (toddler*[Title/Abstract])) OR (kid*[Title/Abstract])) OR (young[Title/Abstract])) OR (pediatric[Title/Abstract])) OR (Adolescents[Title/Abstract])) OR (Adolescent*[Title/Abstract])) OR (Adolescence[Title/Abstract])) OR (Teen*[Title/Abstract])) OR (Teenagers[Title/Abstract])) OR (Teenager[Title/Abstract])) | 658 |
| Scopus  19/8/2023 | ( TITLE-ABS-KEY ( "Non alcoholic Fatty Liver Disease" OR nafld* OR nash* OR non-alcoholic* OR nonalcoholic* OR "Non alcoholic*" OR "Nonalcoholic Fatty Liver*" OR fatty-liver OR "Fatty liver" OR "Nonalcoholic Steatohepatitis" OR "liver steatosis" OR liver-steatosis OR steatohepatitis* OR "hepatic steatosis" OR hepatic-steatosis ) ) AND ( TITLE-ABS-KEY ( child* OR youth OR youngster* OR preschooler* OR toddler* OR kid* OR young OR pediatric OR adolescents OR adolescent* OR adolescence OR teen* OR teenagers OR teenager ) ) AND ( TITLE-ABS-KEY ( "Mediterranean Diet" OR "Mediterranean lifestyle" OR "Mediterranean Diets" OR "cretan diet" OR mediterranean OR "Mediterranean dietary pattern" OR "Mediterranean style diet" OR "Mediterranean diet score" OR "Mediterranean diet index" OR "Mediterranean type diet" OR meddiet OR mediet OR diet OR seafood OR fruit* OR vegetable* OR "red wine" OR legumes OR "olive oil" ) ) | 3,160 |
| Embase  27/8/2023 | #1  **‘non alcoholic fatty liver disease’**:ab,ti OR **nafld***:ab,ti  OR **nash***:ab,ti OR **nonalcoholic***:ab,ti OR **‘non alcoholic*****’**:ab,ti OR **‘nonalcoholic fatty liver*****’**:ab,ti OR **‘fatty liver’**:ab,ti OR **‘nonalcoholic** **steatohepatitis’**:ab,ti OR **‘liver steatosis’:**ab,ti OR **steatohepatitis***:ab,ti OR **‘hepatic steatosis’**:ab,ti | 110,342 |
|  | #2  **‘mediterranean diet’**:ab,ti OR **‘mediterranean lifestyle’**:ab,ti OR **‘mediterranean diets’**:ab,ti OR **‘cretan diet’**:ab,ti OR **mediterranean**:ab,ti OR **‘mediterranean dietary pattern’**:ab,ti OR **‘mediterranean style diet’**:ab,ti OR **‘mediterranean diet score’**:ab,ti OR **‘mediterranean diet index’**:ab,ti OR **‘mediterranean type diet’**:ab,ti OR **meddiet**:ab,ti OR **mediet**:ab,ti OR **diet**:ab,ti OR **seafood**:ab,ti OR **fruit***:ab,ti OR **vegetable***:ab,ti OR **‘red wine’**:ab,ti OR **legumes**:ab,ti OR **‘olive oil’**:ab,ti | 766,606 |
|  | **child***:ab,ti OR **youth**:ab,ti  OR **youngster***:ab,ti OR **preschooler***:ab,ti OR **toddler***:ab,ti OR **kid***:ab,ti OR **young**:ab,ti OR **pediatric**:ab,ti OR **adolescents**:ab,ti OR **adolescent***:ab,ti OR **adolescence**:ab,ti OR **teen***:ab,ti OR **teenagers**:ab,ti OR **teenager**:ab,ti | 3,958,667 |
|  | #4  #1 AND #2 AND #3 | 1,573 |
